# Design, synthesis and evaluation of OA-tacrine hybrids as cholinesterase inhibitors with low neurotoxicity and hepatotoxicity against Alzheimer’s disease

**DOI:** 10.1080/14756366.2023.2192439

**Published:** 2023-03-23

**Authors:** Huali Yang, Hongwei Jia, Minghui Deng, Kaicheng Zhang, Yaoyang Liu, Yang Liu, Maosheng Cheng, Wei Xiao

**Affiliations:** a State Key Laboratory of New-tech for Chinese Medicine Pharmaceutical Process, Jiangsu Kanion Pharmaceutical Co., Ltd, Jiangsu, Lianyungang, China; b Key Laboratory of Structure-Based Drug Design & Discovery of Ministry of Education, School of Pharmaceutical Engineering, Shenyang Pharmaceutical University, China

**Keywords:** Oleanolic acid, tacrine, cholinesterase inhibitors, low-toxicity, Alzheimer’s disease

## Abstract

A series of OA-tacrine hybrids with the alkylamine linker was designed, synthesized, and evaluated as effective cholinesterase inhibitors for the treatment of Alzheimer’s disease (AD). Biological activity results demonstrated that some hybrids possessed significant inhibitory activities against acetylcholinesterase (AChE). Among them, compounds B4 (*h*AChE, IC_50_ = 14.37 ± 1.89 nM; SI > 695.89) and D4 (*h*AChE, IC_50_ = 0.18 ± 0.01 nM; SI = 3374.44) showed excellent inhibitory activities and selectivity for AChE as well as low nerve cell toxicity. Furthermore, compounds B4 and D4 exhibited lower hepatotoxicity than tacrine in cell viability, apoptosis, and intracellular ROS production for HepG2 cells. These properties of compounds B4 and D4 suggest that they deserve further investigation as promising agents for the prospective treatment of AD.

## Graphical Abstract

**Figure:**
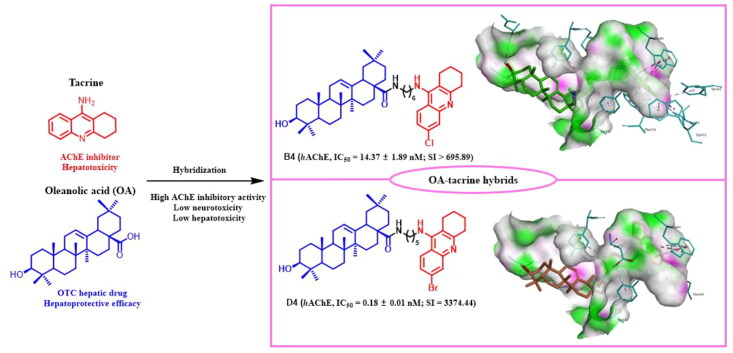

## Introduction

Alzheimer’s disease (AD), an age-related progressive neurodegenerative disorder, afflicts millions of individuals globally in a chronic and fatal way.^1^ It is characterized by memory loss, the decline in language skills, and other cognitive impairments in performing daily activities along with depression.^2^ The pathogenesis of AD is still inconclusive and undefined since it was first reported by Alois Alzheimer in 1907.^3^ Many hypotheses have so far been proposed, including cholinergic neuron system dysfunction,^4^ *β*-amyloid (A*β*) protein deposits,^5^ oxidative stress,^6^ τ-protein hyperphosphorylation and metal dyshomeostasis.^7^^,^^8^

**Figure 1.**
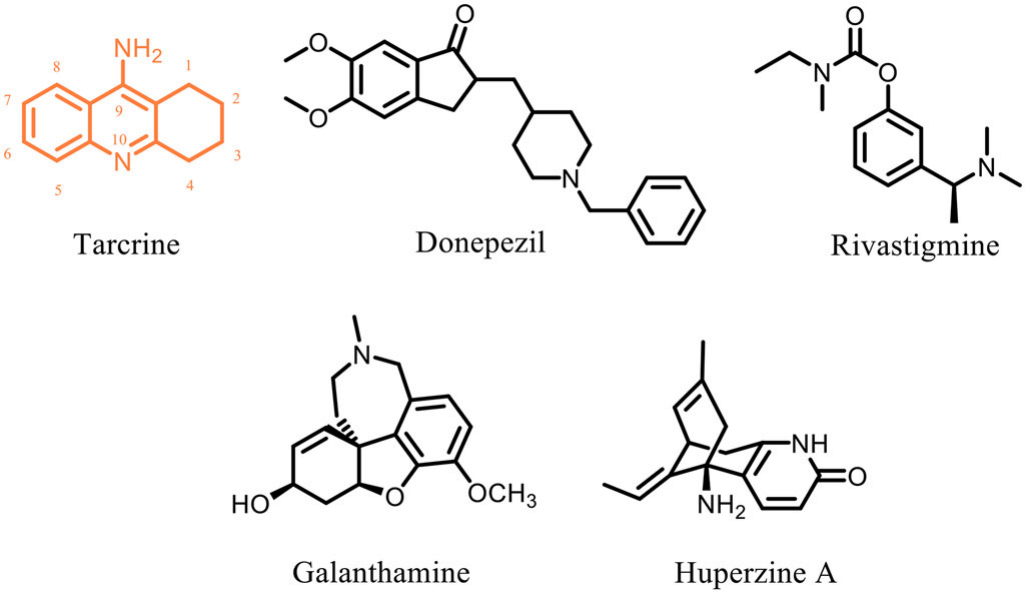
AChEIs are approved by the FDA or CFDA for the treatment of AD.

At present, there are few clinical drugs for the treatment of AD, and they mainly hit a single target. Acetylcholinesterase inhibitors (AChEIs), the first drugs used in the treatment of AD, can enhance the concentration of ACh in the synaptic cleft and improve behavioral disorders in AD patients. Five AChEIs (Figure 1) have been approved for the treatment of AD by the FDA or CFDA: Tacrine, Donepezil, Rivastigmine, Galanthamine and Huperzine A (approved by CFDA).^9^^,^^10^ Tacrine (CAS 321–64-2) is the first AChEI approved for the treatment of mild to moderate AD, but it was regretfully withdrawn by the FDA due to liver toxicity.^1^ Despite favorable AChE inhibitory activity, tacrine has low bioavailability and short half-life, and frequent administration of tacrine is associated with significant hepatotoxicity.^11–13^ Therefore, we focus on searching tacrine derivatives with favorable AChE inhibitory activity and low hepatotoxic side effects, and providing clinically advantageous drugs for the treatment of AD.

**Figure 2.**
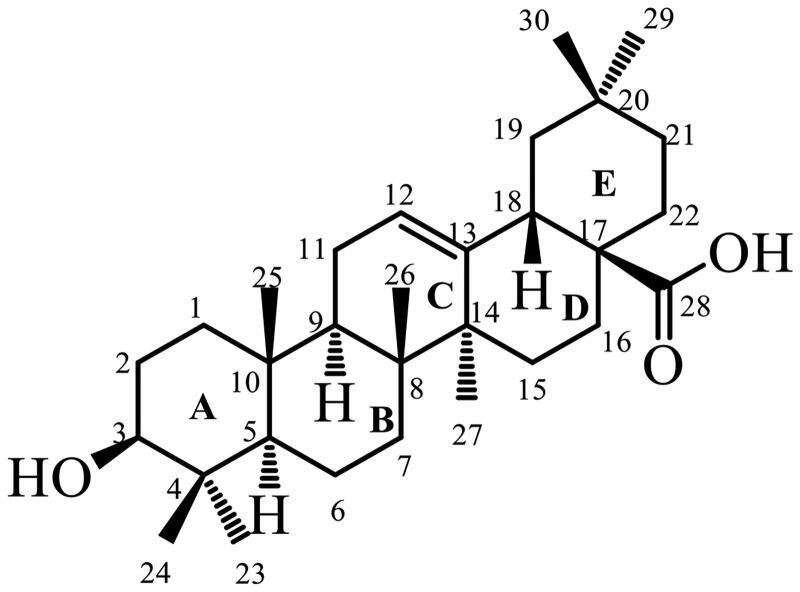
The structure of Oleanolic acid (OA).

The research of traditional Chinese medicine (TCM) in China has a long history and resource advantage. TCM treatment is characterized by integrity and multi-targets. Therefore, starting from natural active ingredients, is very beneficial to the treatment of AD with complex and multiple etiologies. Many natural products have significant therapeutic effects on AD, such as flavonoids, coumarins, alkaloids, phenylpropanoids, triterpenoid saponins, etc. Oleanolic acid (OA, Figure 2), a bioactive natural pentacyclic triterpenoid compound, is present in food and medicinal plants.^14^^,^^15^ OA has been clinically used as an over the counter (OTC) hepatic drug in China for decades.^16^ OA has a significant protective effect against ethanol-induced hepatotoxicity by restoring the levels of hepatotoxic serum marker enzymes in Wistar albino rats.^17^ Besides its hepatoprotective property, pharmacological studies have shown that it has a wide range of effects such as anticancer, anti-osteoporosis, anti-obesity, anti-diabetic, anti-inflammatory, immune-regulatory, and antioxidant effects.^18^^,^^19^ Furthermore, AChE inhibition has been found for derivatives of oleanolic acid.^20^^,^^21^ Anne Loesche et al. synthesized several OA derivatives, screened their inhibitory ability on AChE and BuChE by the Ellman method.^22^ The results show that there are several compounds that have a good inhibitory effect on AChE, and their activities are better than OA. But most of the derivatives, such as OA, had no inhibitory effect on BuChE. Our research group has previously designed and synthesized a series of OA saponin derivatives, and proved that they could improve the cognitive impairment of mice by Aβ-induced dementia mouse model.^23^

**Figure 3.**
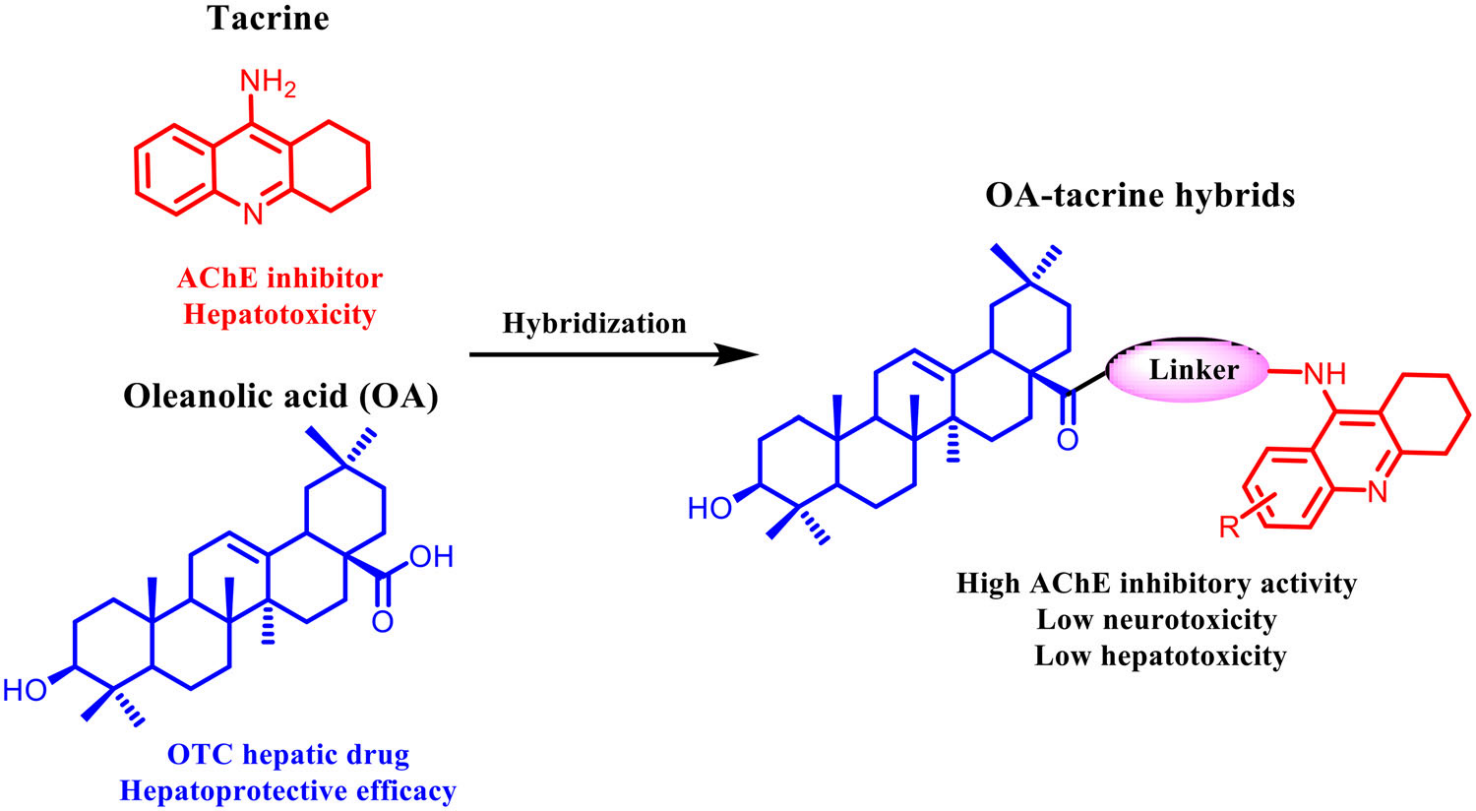
Design strategy of OA-tacrine hybrids.

It was demonstrated by X-ray crystallography that AChE contains two binding sites: the catalytic active site (CAS) and the peripheral anionic site (PAS) connected by a 20 Å deep hydrophobic gorge.^24^ It means that the linker length plays a key role in accommodating ligands inside the AChE narrow gorge. Considering the apparent hepatotoxicity of tacrine and hepatoprotective efficacy of OA, a series of OA-tacrine hybrids with the alkylamine linker (Figure 3) were designed, synthesized, and evaluated for their ChEs inhibitory activity, neurotoxicity, and hepatotoxicity in this study. Meanwhile, computational studies were performed to predict their binding modes in the active pocket and illustrate their exceedingly high affinities.

## Results and discussion

### Chemistry

**Scheme 1.**
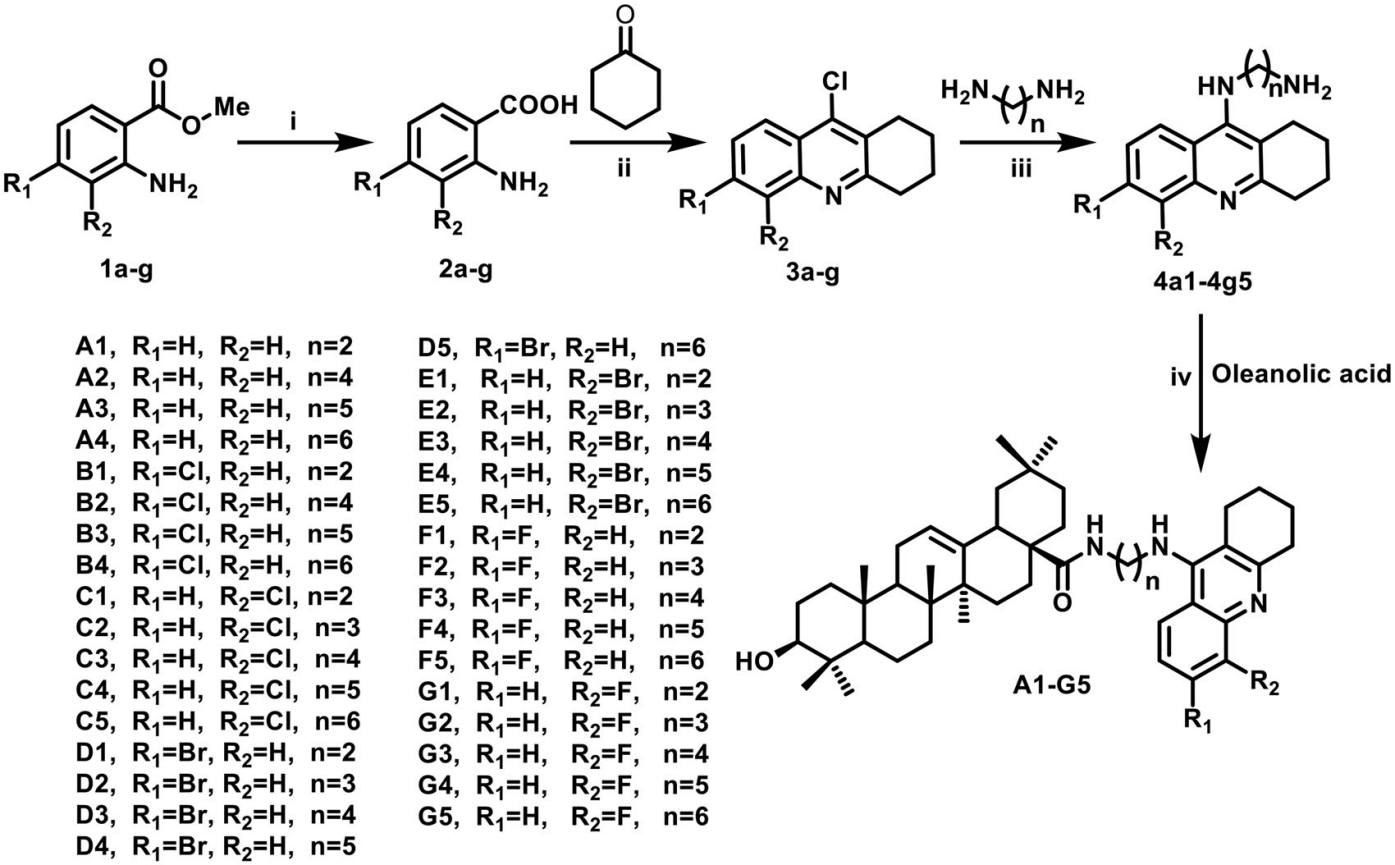
Synthesis of **A1-G5**; reagents and conditions: (i) 20% NaOH aq, 8 h; (ii) cyclohexanone, POCl_3_, reflux, 4 h; (iii) H_2_N(CH_2_)_n_NH_2_, KI, ethylene glycol, reflux, 8 h; (iv) HATU, DIPEA, THF, rt, 3–5 h.

As shown in Scheme 1, tacrine derivatives **3a–g** were obtained from commercial methyl anthranilate by hydrolysis, condensation, and chlorination reactions. And then, target compounds **A1–G5** were synthesized via the reaction of diamine intermediates **4a1-4g5** and the commercial OA. The structures of all newly synthesized compounds were confirmed by various methods of spectroscopic analysis, such as ^1^H NMR, ^13^C NMR, and ESI-MS.

### Biological assays

#### Inhibitory activities against hAChE and hBuChE

The *h*AChE and *h*BuChE inhibitory activities of OA-tacrine hybrids were measured by Ellman’s method using tacrine as reference compound.^25^ Acetylcholinesterase Activity Assay Kit and Butyrylcholinesterase Activity Assay Kit purchased form sigma-Aldrich. The corresponding IC_50_ values and selectivity index (SI) values are shown in Table 1. As shown in Table 1, the type and location of substituents from benzene ring of tacrine, as well as the linker length between OA and tacrine played a significant role in determining the ChEs inhibitory activity and selectivity. Comparing the IC_50_ values of B1-4, D1-5, and F1-5, compounds with Cl or Br substitution from the benzene ring of tacrine performed much better activities than that with F substitution in *h*AChE inhibition. Among them, compound D4 (*h*AChE, IC_50_ = 0.18 ± 0.007 nM; SI = 3374.44) with Br substitution showed the most potent inhibitory activity and selectivity for *h*AChE, which was stronger than the reference compound tacrine (*h*AChE, IC_50_ = 305.78 ± 103.44 nM; SI = 0.16). Compounds B4 (*h*AChE, IC_50_ = 14.37 ± 1.89 nM; SI > 695.89) and D4 with R_1_-position substitution had more potent *h*AChE inhibitory activities than compounds C5 (*h*AChE, IC_50_ = 2721.68 ± 25.83 nM; SI > 3.67) and E4 (*h*AChE, IC_50_ >10^4^ nM) with R_2_-position substitution. Compounds G1-5 with R_2_-position substitution had more potent *h*BuChE inhibitory activities. Among them, G1 (*h*BuChE, IC_50_ = 153.79 ± 94.42 nM) with F substitution showed the most potent inhibitory activity and selectivity for *h*BuChE.

**Table 1.** *h*AChE and *h*BuChE inhibitory activities of target compounds **A1-G5**.

Compound	*n*	IC_50_ (nM)*^a^*		SI*^c^*
		*h*AChE*^b^*	*h*BuChE*^b^*	
**Tacrine**	–	305.78 ± 103.44	56.75 ± 4.32	0.19
**A1**	2	>10^4^	>10^4^	–
**A2**	4	444.84 ± 78.72	2347.90 ± 84.55	5.28
**A3**	5	774.79 ± 280.43	2780.52 ± 60.71	3.59
**A4**	6	>10^4^	2688.21 ± 40.15	<0.27
**B1**	2	147.73 ± 61.37	>10^4^	>67.69
**B2**	4	954.15 ± 184.42	>10^4^	>10.48
**B3**	5	460.35 ± 20.11	>10^4^	>21.72
**B4**	6	14.37 ± 1.89	>10^4^	>695.89
**C1**	2	>10^4^	>10^4^	–
**C2**	3	>10^4^	>10^4^	–
**C3**	4	>10^4^	>10^4^	–
**C4**	5	>10^4^	>10^4^	–
**C5**	6	2721.68 ± 25.83	>10^4^	>3.67
**D1**	2	>10^4^	>10^4^	–
**D2**	3	>10^4^	>10^4^	–
**D3**	4	1850.99 ± 504.49	>10^4^	>5.40
**D4**	5	0.18 ± 0.01	607.40 ± 83.13	3374.44
**D5**	6	>10^4^	>10^4^	–
**E1**	2	>10^4^	>10^4^	–
**E2**	3	>10^4^	>10^4^	–
**E3**	4	>10^4^	>10^4^	–
**E4**	5	>10^4^	>10^4^	–
**E5**	6	>10^4^	>10^4^	–
**F1**	2	>10^4^	>10^4^	–
**F2**	3	>10^4^	>10^4^	–
**F3**	4	>10^4^	>10^4^	
**F4**	5	>10^4^	2058 ± 66.67	<0.21
**F5**	6	4213.02 ± 101.22	3994.10 ± 1138.30	0.95
**G1**	2	>10^4^	198.72 ± 15.11	<0.02
**G2**	3	>10^4^	921.03 ± 58.71	<0.09
**G3**	4	>10^4^	955.42 ± 67.55	<0.10
**G4**	5	>10^4^	1603.67 ± 90.42	<0.16
**G5**	6	>10^4^	3809.40 ± 1036.90	<0.38

*^a^*IC_50_: 50% inhibitory concentration (mean ± SD of three experiments).

*^b^h*AChE from human erythrocytes and *h*BuChE from human serum were used.

*^c^*SI means selectivity index, *h*BuChE/*h*AChE.

#### Molecular docking studies

**Figure 4.**
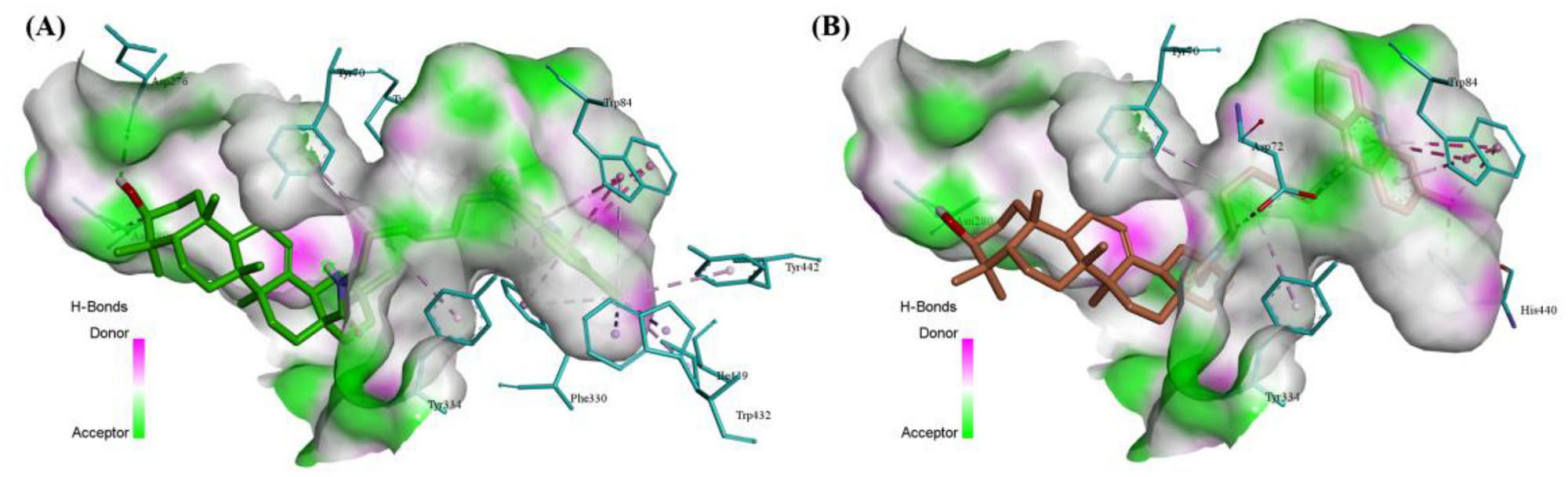
Docking study of compounds B4 (A：colored green) and D4 (B：colored orange) with AChE (PDB code 2CKM). The semi-transparent surface represents the active site cavity.

To gain insights into the binding patterns of these compounds with the AChE enzymes, the molecular modeling study based on AChE (PDB code: 2CKM) was performed using the docking program, AutoDock 4.2 package with Discovery Studio 2.0. As shown in Figure 4, compounds B4 and D4 could fit into the active-site gorge of the enzyme and simultaneously interact with the PAS and CAS of AChE. The tacrine fragment of target molecules can penetrate into the CAS binding site, and the oleanolic acid skeleton is located at the PAS binding site. The alcohol hydroxyl groups of compounds B4 and D4 formed hydrogen bonds with the oxygen atoms of Asp276 and Asn280, respectively. The amino group of the tacrine fragment of compound D4 can also form a hydrogen bond with Asp72, which may be the reason why D4 has better AChE inhibitory activity. In addition, the π–π stacking interactions were formed between the tacrine fragment and Trp84. Based on the above discussion, it was evident that hydrogen bonds and π–π stacking interactions were important for binding patterns of the potential compounds with AChE active site.

#### Toxicity studies

##### Effect of cell viabilities in SH-SY5Y and HepG2 cells

**Figure 5.**
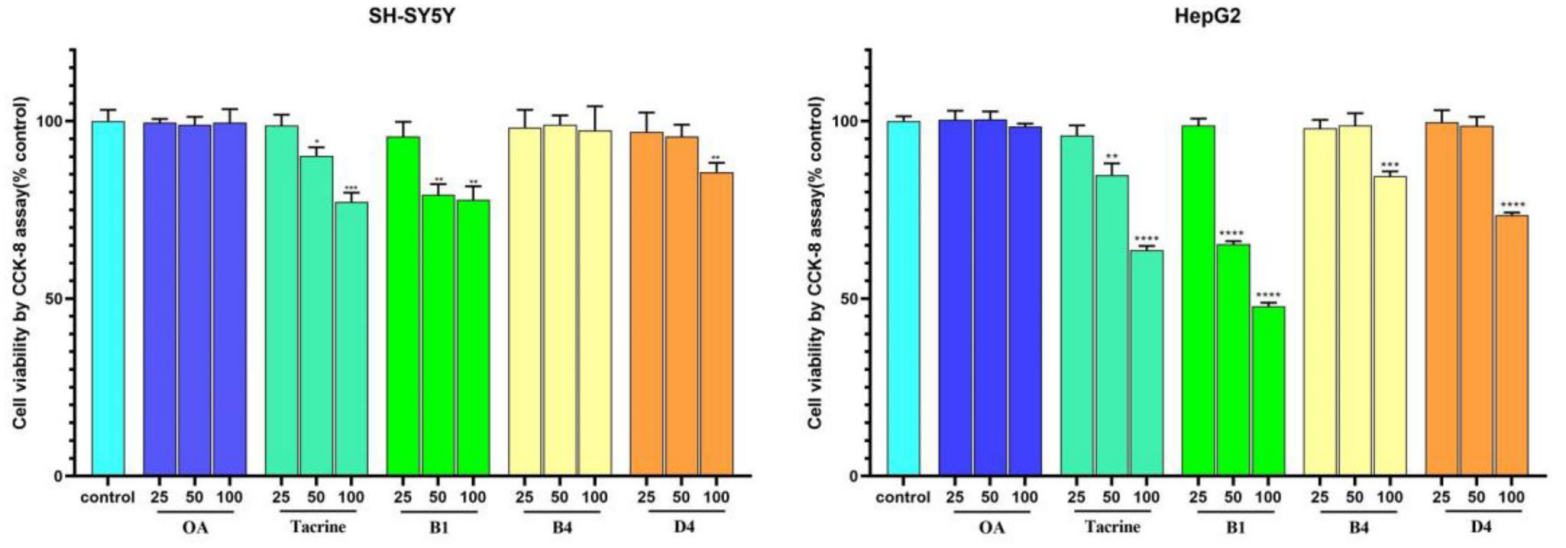
Effects of various concentrations of compounds on cell viability in SH-SY5Y cells and HepG2 cells after treatment for 24 h. Cell viability was measured by CCK-8 assay. Data were shown as mean ± SD of three independent experiments.

The safety is extraordinarily important for the CNS drugs, so the potential toxicity effect of OA-tacrine hybrids was investigated on SH-SY5Y cells and HepG2 cells by Cell Counting Kit-8 (CCK-8) assay. Compounds B1, B4, and D4, which had more potent *h*AChE inhibitory activities, were selected as representative compounds to be evaluated for potential cytotoxic effects at the concentrations of 25, 50, 100 μM. OA and tacrine were used as the reference. As shown in Figure 5, tacrine and compound B1 were cytotoxic to both SH-SY5Y cells and HepG2 cells at concentrations of 50–100 μM, indicating that they had obvious neurotoxicity and hepatotoxicity. Compounds B4 and D4 showed little effect on the SH-SY5Y cell viability at the concentration of 50 μM. And the HepG2 cell viability rates of compounds B4 and D4 at the concentration of 50 μM were close to that of the control group. The data showed that compounds B4 and D4 with low neurotoxicity and hepatotoxicity might be used to develop promising drug candidates for the therapy of treating AD.

##### Effect of apoptosis in HepG2 cells

**Figure 6.**
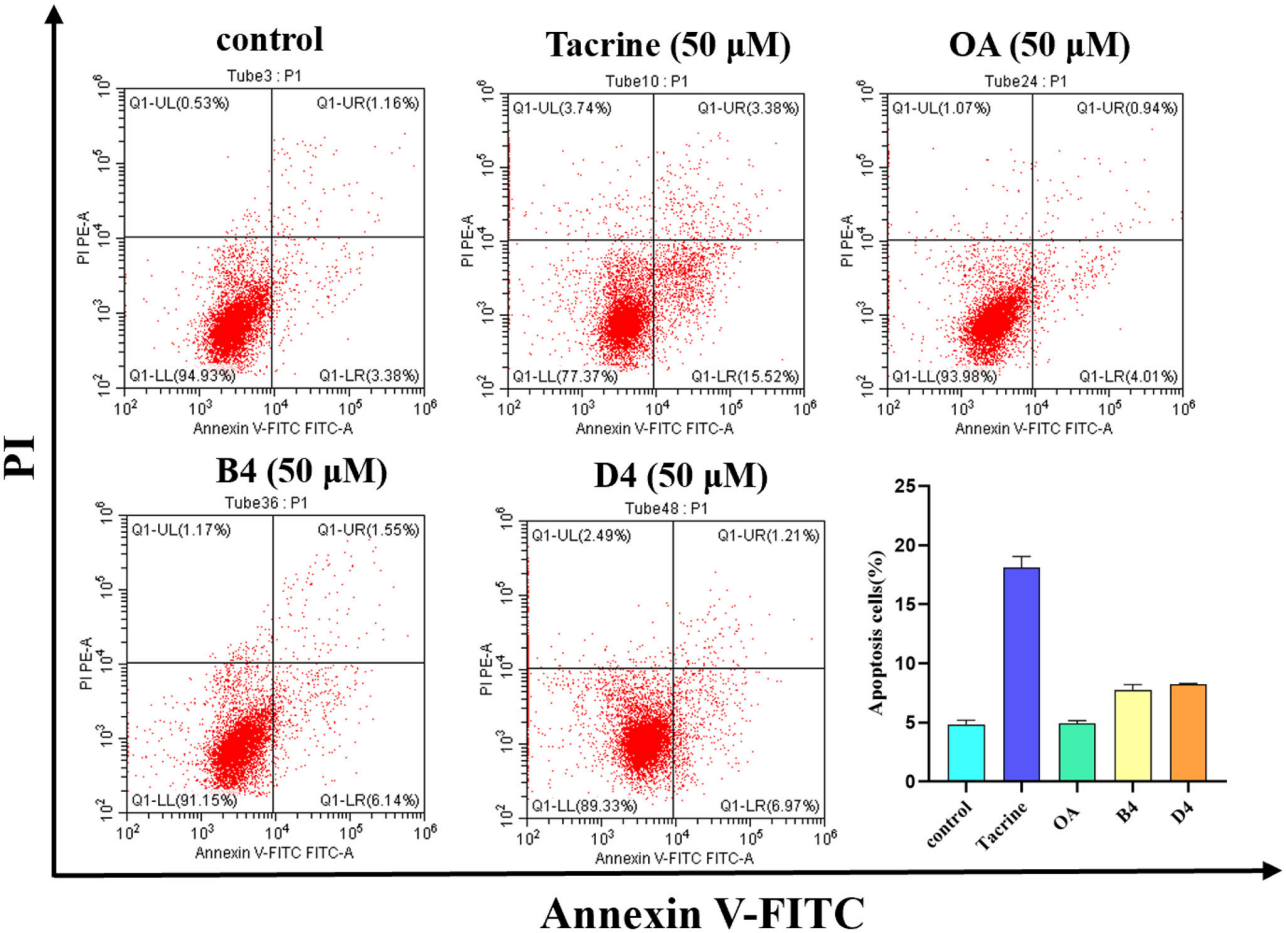
The effect of compounds B4 and D4 on apoptosis in HepG2 cells. Apoptotic cells detected by flow cytometry after AnnexinV and PI double staining.

As mentioned above, hepatotoxicity is a major side effect for tacrine. Considering hepatoprotective efficacy of OA, the strategy adopted for the design of OA-tacrine hybrids in this work. To further investigate the hepatotoxicity of hybrids, AnnexinV/PI double staining method was used to evaluate the effects of compounds B4 and D4 on cell apoptosis at the concentration of 50 μM. As shown in Figure 6, the percentage of apoptotic cells increased from 4.34% (control group) to 18.90% (Tacrine group). Compared with the tacrine-treated group, the groups treated compounds B4 and D4 (7.69% and 8.18%, respectively) showed significantly reduced percentages of apoptotic cells. This result indicated that compounds B4 and D4 have been proposed as potential inhibitors of cholinesterase with low hepatotoxicity.

##### Effect of ROS production in HepG2 cells

**Figure 7.**
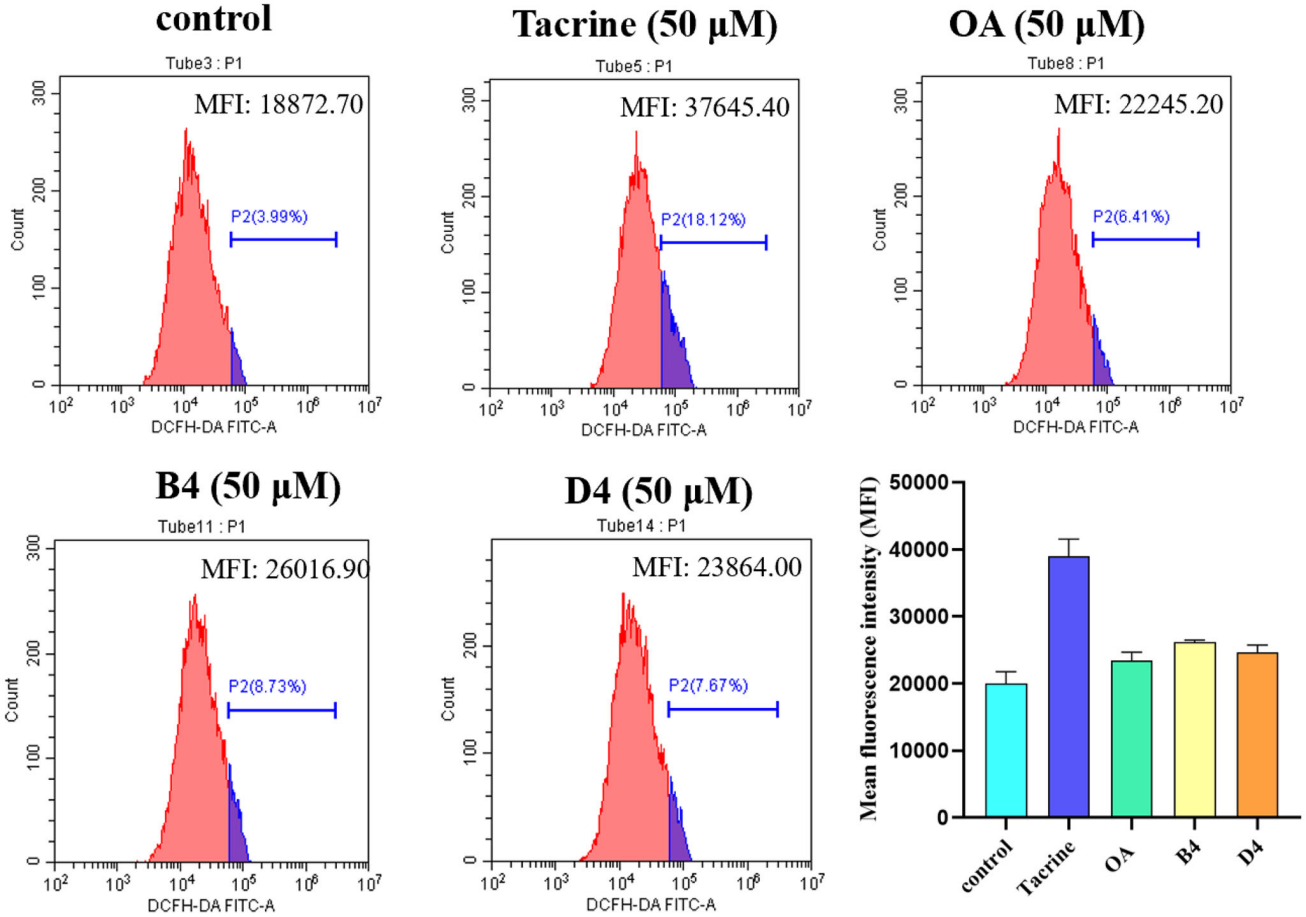
The effect of compounds B4 and D4 on intracellular ROS production in HepG2 cells. The percentage of cells for ROS was stained with DCFH-DA and the stained cells were immediately determined by flow cytometry.

Studies have shown that the hepatotoxicity induced by tacrine mainly resulted in elevating the levels liver transaminase, decreasing albumin concentration and inducing ROS in hepatocyte ^26^^,^.^27^ Therefore, the effects of compounds B4 and D4 on ROS production in HepG2 cells at the concentration of 50 μM was evaluated by flow cytometry analysis. As shown in Figure 7, in comparison to the control group, tacrine at the concentration of 50 μM caused significant hepatotoxicity. While treated with compounds B4 and D4, the intracellular ROS levels were significantly lower than that in the tacrine group. This result further indicated that compounds B4 and D4 have low hepatotoxicity.

## Conclusions

In summary, a series of OA-tacrine hybrids with the alkylamine linker was designed and synthesized as effective ChEs inhibitors against AD. Among the synthesized compounds, compounds B4 (*h*AChE, IC_50_ = 14.37 ± 1.89 nM; SI > 695.89) and D4 (*h*AChE, IC_50_ = 0.11 ± 0.48 nM; SI = 5521.82) showed more potent inhibitory activities and selectivities for *h*AChE, which were stronger than the reference compound tacrine (*h*AChE, IC_50_ = 305.78 ± 103.44 nM; SI = 0.16). The molecular modeling study has been done to gain insights into the binding patterns of these compounds with the AChE enzymes. More importantly, the cytotoxicity tests showed that compounds B4 and D4 had low cytotoxic activity toward SH-SY5Y cells and HepG2 cells. Besides, these compounds showed lower hepatotoxicity in HepG2 cells by inducing less cell apoptosis and intracellular ROS than tacrine. These properties highlighted that compounds B4 and D4 with high AChE inhibitory activities, low neurotoxicity and hepatotoxicity could be considered as potential agents for the development of anti-AD drugs.

## Experimental section

### Chemistry

All chemicals (reagent grade) used were purchased from Sino pharm Chemical Reagent Co., Ltd. (China). Reaction progress was monitored using analytical thin layer chromatography (TLC) on precoated silica gel GF254 (Qingdao Haiyang Chemical Plant, Qing-Dao, China) plates and the spots were detected under UV light (254 nm). Column chromatography was performed on silica gel (90–150 *μ*m; Qingdao Marine Chemical Inc.). ^1^H NMR and ^13^C NMR spectra were measured on a Bruker ACF-500 spectrometer at 25 °C and referenced to TMS. Chemical shifts are reported in ppm (*δ*) using the residual solvent line as internal standard. Splitting patterns are designed as s, singlet; d, doublet; t, triplet; m, multiplet. Mass spectra were obtained on a MS Agilent 1100 Series LC/MSD Trap mass spectrometer (ESI-MS).

#### General method for preparation of tacrine derivatives 3a–g

Commercial methyl anthranilate **1a–g** (5.0 mmol) were dissolved in 20% NaOH aqueous solution stirred for 5 h, adjusted the pH to 2, filtered and dried to obtain anthranilic acid **2a–g.** Then under ice bath (0 °C), slowly add compound **2a–g** (5.0 mmol) and cyclohexanone (6.0 mmol) to POCl_3_ (15 ml). 5 min later, removed the ice bath and stirred at 90 °C with refluxing for 5 h, adjusted the pH to 10, extracted with dichloromethane and subjected to column chromatography (petroleum ether: ethyl acetate = 10: 1).

##### 9-chloro-1,2,3,4-tetrahydroacridine 3a

White solid (0.89 g, 4.1 mmol), yield 82.00%. ^1^H NMR (600 MHz, Chloroform- *d*) δ 8.12 (dd, *J* = 8.5, 1.4 Hz, 1H), 7.94 (dd, *J* = 8.4, 1.2 Hz, 1H), 7.75 (ddd, *J* = 8.4, 6.8, 1.4 Hz, 1H), 7.64 (ddd, *J* = 8.2, 6.8, 1.2 Hz, 1H), 3.04 (h, *J* = 2.6, 2.1 Hz, 2H), 2.96 (td, *J* = 5.6, 4.6, 2.2 Hz, 2H), 1.88 (p, *J* = 3.4 Hz, 4H); ESI-MS *m/z* 218.1[M + H]^+^.

##### 6,9-dichloro-1,2,3,4-tetrahydroacridine 3b

Yellow solid (0.98 g, 3.9 mmol), yield 78.03%. ^1^H NMR (600 MHz, Chloroform -d) δ 8.08 (dd, *J* = 8.5, 1.3 Hz, 1H), 7.76 (dd, *J* = 7.4, 1.3 Hz, 1H), 7.42 (dd, *J* = 8.5, 7.4 Hz, 1H), 3.20 (t, *J* = 6.1 Hz, 2H), 3.00 (t, *J* = 6.2 Hz, 2H), 1.95 (qd, *J* = 4.5, 1.9 Hz, 4H); ESI-MS m/z 252.1[M + H]+.

##### 5,9-dichloro-1,2,3,4-tetrahydroacridine 3c

Yellow solid (0.93 g, 3.7 mmol), yield 74.10%. ^1^H NMR (600 MHz, Chloroform- *d*) δ 7.29 − 7.23 (m, 1H), 7.06 (d, *J* = 6.9 Hz, 1H), 6.93 (td, *J* = 8.5, 2.6 Hz, 1H), 2.78 (d, *J* = 11.5 Hz, 2H), 2.15 − 2.06 (m, 2H), 1.95 − 1.88 (m, 2H), 1.41 (dd, *J* = 11.1, 3.4 Hz, 2H); ESI-MS *m/z* 252.1[M + H]^+^.

##### 6-bromo-9-chloro-1,2,3,4-tetrahydroacridine 3d

Yellow solid (1.27 g, 4.3 mmol), yield 86.03%. ^1^H NMR (600 MHz, Chloroform- *d*) δ 8.18 (d, *J* = 1.9 Hz, 1H), 8.01 (d, *J* = 8.9 Hz, 1H), 7.60 (dd, *J* = 8.9, 2.0 Hz, 1H), 3.12 (t, *J* = 6.0 Hz, 2H), 2.98 (t, *J* = 6.2 Hz, 2H), 1.95 (h, *J* = 4.4, 3.1 Hz, 4H); ESI-MS *m/z* 295.9[M + H]^+^.

##### 5-bromo-9-chloro-1,2,3,4-tetrahydroacridine 3e

Yellow solid (1.22 g, 4.2 mmol), yield 83.97%. ^1^H NMR (600 MHz, Chloroform- *d*) δ 8.15 (dd, *J* = 8.4, 1.3 Hz, 1H), 7.99 (dd, *J* = 7.5, 1.3 Hz, 1H), 7.36 (t, *J* = 7.9 Hz, 1H), 3.24 − 3.17 (m, 2H), 3.07 − 2.99 (m, 2H), 1.96 (tt, *J* = 6.1, 3.2 Hz, 4H); ESI-MS *m/z* 295.9[M + H]^+^.

##### 9-chloro-6-fluoro-1,2,3,4-tetrahydroacridine 3f

Brown solid (0.82 g, 3.5 mmol), yield 70.02%. ^1^H NMR (600 MHz, Chloroform- *d*) δ 8.15 (dd, *J* = 9.2, 6.0 Hz, 1H), 7.60 (dd, *J* = 10.0, 2.6 Hz, 1H), 7.31 (td, *J* = 8.7, 2.6 Hz, 1H), 3.10 (t, *J* = 6.1 Hz, 2H), 2.99 (t, *J* = 6.1 Hz, 2H), 1.94 (dq, *J* = 8.4, 5.1, 4.6 Hz, 4H); ESI-MS *m/z* 236.1[M + H]^+^.

##### 9-chloro-5-fluoro-1,2,3,4-tetrahydroacridine 3 g

Brown solid (0.76 g, 3.2 mmol), yield 64.03%. ^1^H NMR (600 MHz, Chloroform- *d*) δ 7.93 (dd, *J* = 8.5, 1.2 Hz, 1H), 7.45 (td, *J* = 8.1, 5.0 Hz, 1H), 7.35 (ddd, *J* = 10.5, 7.7, 1.2 Hz, 1H), 3.18 (t, *J* = 6.1 Hz, 2H), 3.02 (t, *J* = 6.1 Hz, 2H), 1.95 (qt, *J* = 4.6, 2.3 Hz, 4H); ESI-MS *m/z* 236.1[M + H]^+^.

#### General method for preparation of target compounds A1–G5

Tacrine derivatives **3a–g** (1.0 mmol), diaminoalkanes (6.0 mmol) of various lengths (*n* = 2,3,4,5,6) and KI (0.1 mmol) were mixed and dissolved in ethylene glycol (10 ml). Heat up to 160 °C and stirred for 8h, then extracted with dichloromethane and concentrated to obtain brown oil diamine intermediates **4a1–4g5**. Meanwhile, oleanolic acid (1.1 mmol) was dissolved in THF with HATU (1.1 mmol) and DIPEA (2.2 mmol) at room temperature and stirred for 0.5h. Then added brown oil diamine intermediates **4a1–4g5** to them. About 3-5h later, concentrated to column chromatography (dichloromethane: Methanol = 20: 1).

##### N-(2-((1,2,3,4-tetrahydroacridin-9-yl)amino)ethyl)-olean-12-en-28- amide A1

White solid (0.22 g, 0.32 mmol), yield 32.33%. ^1^H NMR (600 MHz, Chloroform-*d*) δ 12.18 (s, 1H, olean amide), 8.23 (d, *J* = 8.6 Hz, 1H, H8-Tacrine), 7.87 (d, *J* = 8.5 Hz, 1H, H5-Tacrine), 7.83 (d, *J* = 8.3 Hz, 1H, H6-Tacrine), 7.69 (t, *J* = 7.7 Hz, 1H, H7-Tacrine), 7.51 − 7.46 (m, 1H, -NH-), 5. 46 (t, *J* = 3.6 Hz, 1H, CH = C), 3.97 (t, *J* = 6.9 Hz, 2H), 3.64 (heptd, *J* = 6.6, 4.2 Hz, 4H), 3.44 (dd, *J* = 13.6, 6.8 Hz, 1H), 3.26 − 3.18 (m, 2H), 3.12 (qd, *J* = 7.4, 4.2 Hz, 4H), 3.02 (t, *J* = 6.2 Hz, 2H), 2.63 (t, *J* = 5.9 Hz, 2H), 2.59 (dd, *J* = 12.3, 4.0 Hz, 1H), 2.08 − 1.98 (m, 2H), 1.95 − 1.90 (m, 2H), 1.86 (dd, *J* = 9.1, 5.4 Hz, 4H), 1.76 (d, *J* = 13.4 Hz, 2H), 1.73 − 1.66 (m, 4H), 1.58 − 1.52 (m, 4H), 1.44 (s, 2H), 1.25 (t, *J* = 4.0 Hz, 4H), 1.14 (s, 3H, CH3), 0.97 (s, 3H, CH3), 0.90 (d, *J* = 2.7 Hz, 6H, 2CH3), 0.81 (s, 3H, CH3), 0.75 (s, 3H, CH3), 0.70 (s, 3H, CH3); ^13^C NMR (151 MHz, Chloroform-*d*) δ 156.22, 150.44, 143.38, 138.33, 132.82 (C6-Tacrine), 129.84 (C5-Tacrine), 129.80 (C7-Tacrine), 125.35 (C8-Tacrine), 124.72, 123.72, 119.79, 115.50, 111.43, 54.94, 54.84, 47.77, 47.32, 46.36, 46.33, 43.00, 41.80, 41.51, 39.28, 38.64, 38.31, 36.80, 33.90, 32.84, 32.21, 30.52, 29.63, 28.19, 27.98, 27.60, 27.13, 25.72, 23.39, 23.36, 21.74, 20.59, 18.40, 18.15, 16.97, 16.85, 15.50, 15.14, 12.47; ESI-MS *m/z* 680.5 [M + H]^+^.

##### N-(4-((1,2,3,4-tetrahydroacridin-9-yl)amino)butyl)-olean-12-en-28- amide A2

White solid (0.25 g, 0.35 mmol), yield 35.29%. ^1^H NMR (600 MHz, Chloroform-*d*) δ 10.74 (s, 1H, olean amide), 8.28 (d, *J* = 8.7 Hz, 1H, 1H, H8-Tacrine), 8.19 (s, 1H, -NH-), 7.82 (dd, *J* = 8.6, 1.2 Hz, 1H, H5-Tacrine), 7.69 (ddd, *J* = 8.3, 6.9, 1.1 Hz, 1H, H7-Tacrine), 7.46 (ddd, *J* = 8.5, 7.0, 1.3 Hz, 1H, H6-Tacrine), 5.44 (t, *J* = 3.6 Hz, 1H, CH = C), 4.16 − 4.07 (m, 2H), 3.85 − 3.59 (m, 6H), 3.19 (qd, *J* = 7.4, 4.4 Hz, 4H), 3.03 (t, *J* = 6.2 Hz, 2H), 2.74 − 2.66 (m, 2H), 2.66 − 2.60 (m, 1H), 2.08 − 2.00 (m, 2H), 1.95 − 1.91 (m, 2H), 1.88 (tt, *J* = 8.8, 4.7 Hz, 4H), 1.74 (t, *J* = 12.9 Hz, 6H), 1.59 − 1.56 (m, 2H), 1.54 − 1.51 (m, 2H), 1.23 − 1.18 (m, 4H), 1.14 (s, 3H, CH_3_), 0.96 (s, 3H, CH_3_), 0.90 (d, *J* = 3.3 Hz, 6H, 2CH_3_), 0.78 (s, 3H, CH_3_), 0.74 (s, 3H, CH_3_), 0.55 (s, 3H, CH_3_); ^13^C NMR (151 MHz, Chloroform-*d*) δ 182.29, 157.03, 143.70(C6-Tacrine), 132.95(C5-Tacrine), 125.20(C7-Tacrine), 123.50(C8-Tacrine), 119.49, 115.56, 111.43, 55.72, 47.31, 46.44, 46.28, 43.67, 41.81, 41.61, 39.67, 39.20, 38.63, 38.30, 36.80, 33.93, 32.87, 32.61, 32.13, 30.60, 29.62, 29.58, 29.24, 28.22, 27.94, 27.16, 27.13, 26.98, 23.49, 23.41, 23.33, 21.89, 20.69, 18.48, 18.06, 17.02, 16.54, 15.41, 15.11, 14.05, 12.59; ESI-MS *m/z* 708.5 [M + H]^+^.

##### N-(5-((1,2,3,4-tetrahydroacridin-9-yl)amino)pentyl)-olean-12-en-28-amide A3

White solid (0.37 g, 0.51 mmol), yield 51.21%. ^1^H NMR (600 MHz, Chloroform-*d*) δ 11.30 (s, 1H, olean amide), 8.25 (d, *J* = 8.7 Hz, 1H, H8-Tacrine), 7.82 (dd, *J* = 8.6, 1.2 Hz, 1H, H5-Tacrine), 7.72 (ddd, *J* = 8.3, 7.0, 1.1 Hz, 1H, H7-Tacrine), 7.49 (ddd, *J* = 8.4, 6.9, 1.2 Hz, 1H, H6-Tacrine), 6.15 (t, *J* = 5.8 Hz, 1H, -NH-), 5.38 (t, *J* = 3.7 Hz, 1H, CH = C), 3.92 (h, *J* = 6.4 Hz, 2H), 3.71 (pd, *J* = 6.6, 4.2 Hz, 1H), 3.35 (dq, *J* = 13.7, 7.1 Hz, 1H), 3.24 − 3.17 (m, 2H), 3.06 − 3.02 (m, 1H), 3.00 (q, *J* = 6.3, 4.5 Hz, 2H), 2.63 (t, *J* = 6.2 Hz, 2H), 2.55 (dd, *J* = 13.2, 4.3 Hz, 1H), 1.93 (dt, *J* = 6.9, 3.4 Hz, 2H), 1.91 − 1.87 (m, 4H), 1.75 (t, *J* = 13.4 Hz, 1H), 1.68 − 1.58 (m, 5H), 1.57 − 1.51 (m, 7H), 1.43 (s, 1H), 1.43 − 1.38 (m, 8H), 1.36 (d, *J* = 6.3 Hz, 2H), 1.27 − 1.22 (m, 2H), 1.20 − 1.18 (m, 1H), 1.15 (s, 3H, CH_3_), 0.98 (s, 3H, CH_3_), 0.90 (s, 6H, 2CH_3_), 0.86 (s, 3H, CH_3_), 0.75 (d, *J* = 3.8 Hz, 6H, 2CH_3_); ^13^C NMR (151 MHz, Chloroform-*d*) δ 156.29, 150.06, 144.45, 138.19, 132.99, 125.31(C6-Tacrine), 124.95(C5-Tacrine), 122.87(C7-Tacrine), 119.57(C8-Tacrine), 115.37, 111.32, 55.62, 54.93, 48.03, 47.35, 46.54, 46.15, 43.58, 41.98, 41.91, 39.22, 38.98, 38.62, 38.30, 36.81, 33.99, 32.85, 32.63, 32.27, 30.58, 30.20, 29.01, 28.21, 27.98, 27.12, 26.96, 25.95, 25.63, 25.58, 23.68, 23.44, 23.37, 22.88, 21.61, 20.52, 18.40, 18.14, 16.88, 15.49, 15.20, 12.68; ESI-MS *m/z* 722.5 [M + H]^+^.

##### N-(6-((1,2,3,4-tetrahydroacridin-9-yl)amino)hexyl)-olean-12-en-28-amide A4

White solid (0.21 g, 0.27 mmol), yield 27.51%. ^1^H NMR (600 MHz, Chloroform-*d*) δ 11.47 (s, 1H, olean amide), 8.24 (d, *J* = 8.7 Hz, 1H, H8-Tacrine), 7.90 (d, *J* = 8.5 Hz, 1H, H5-Tacrine), 7.73 − 7.68 (m, 1H, H7-Tacrine), 7.51 − 7.44 (m, 1H, H6-Tacrine), 6.07 (s, 1H, -NH-), 5.38 (t, *J* = 3.7 Hz, 1H, CH = C), 3.93 (q, *J* = 6.0 Hz, 2H), 3.40 (dq, *J* = 13.5, 6.8 Hz, 1H), 3.21 (dd, *J* = 11.3, 4.5 Hz, 1H), 3.04 (t, *J* = 6.4 Hz, 2H), 2.61 (t, *J* = 6.2 Hz, 2H), 2.56 − 2.50 (m, 1H), 2.00 (dd, *J* = 13.5, 3.6 Hz, 1H), 1.92 (d, *J* = 6.6 Hz, 2H), 1.90 − 1.86 (m, 4H), 1.75 (t, *J* = 13.4 Hz, 2H), 1.70 − 1.64 (m, 4H), 1.60 (d, *J* = 4.3 Hz, 2H), 1.59 − 1.57 (m, 2H), 1.55 (d, *J* = 3.4 Hz, 2H), 1.54 − 1.50 (m, 2H), 1.47 (t, *J* = 7.4 Hz, 2H), 1.45 (d, *J* = 3.7 Hz, 1H), 1.36 (d, *J* = 4.3 Hz, 1H), 1.33 (dd, *J* = 13.3, 3.8 Hz, 2H), 1.27 − 1.24 (m, 6H, 2CH_3_), 1.23 (d, *J* = 3.0 Hz, 1H), 1.19 (d, *J* = 3.8 Hz, 2H), 1.15 (s, 3H, CH_3_), 1.08 − 1.00 (m, 2H), 0.98 (s, 3H, CH_3_), 0.95 (d, *J* = 3.2 Hz, 1H), 0.89 (d, *J* = 3.0 Hz, 6H, 2CH_3_), 0.86 (s, 3H, CH_3_), 0.75 (d, *J* = 7.0 Hz, 5H); ^13^C NMR (151 MHz, Chloroform-*d*) δ 178.95, 156.39, 150.62, 144.68, 138.30, 133.14, 125.45(C6-Tacrine), 124.81(C5-Tacrine), 122.95(C7-Tacrine), 120.00(C8-Tacrine), 115.53, 111.28, 55.03, 53.43, 50.87, 48.73, 47.46, 46.71, 46.33, 42.13, 42.04, 39.35, 38.87, 38.73, 38.40, 36.92, 34.11, 32.95, 32.73, 32.33, 31.92, 31.90, 30.70, 30.01, 29.77, 29.69, 29.65, 29.60, 29.55, 29.51, 29.48, 29.36, 29.34, 29.30, 29.23, 28.31, 28.08, 27.26, 27.20, 27.09, 25.74, 24.03, 23.83, 23.56, 23.48, 23.21, 22.68, 21.73, 20.59, 18.25, 16.99, 15.57, 15.32, 14.12; ESI-MS *m/z* 736.5 [M + H]^+^.

##### N-(2-((6-chloro-1,2,3,4-tetrahydroacridin-9-yl)amino)ethyl)-olean-12-en-28- amide B1

White solid (0.22 g, 0.1 mmol), yield 30.79%. ^1^H NMR (600 MHz, Chloroform-*d*) δ 7.99 − 7.95 (m, 1H, H5-Tacrine), 7.92 (d, *J* = 9.1 Hz, 1H, H8-Tacrine), 7.28 (d, *J* = 8.9 Hz, 1H, H7-Tacrine), 6.00 (t, *J* = 5.3 Hz, 1H -NH-), 5.30 (t, *J* = 3.6 Hz, 1H, CH = C), 3.57 (t, *J* = 6.6 Hz, 2H), 3.43 − 3.36 (m, 1H), 3.20 (dd, *J* = 11.3, 4.4 Hz, 1H), 3.07 (s, 2H), 3.04 (qd, *J* = 7.6, 6.6, 4.9 Hz, 1H), 2.68 (d, *J* = 5.8 Hz, 2H), 2.47 (dd, *J* = 13.3, 4.4 Hz, 1H), 1.96 (td, *J* = 13.7, 3.8 Hz, 2H), 1.91 (h, *J* = 4.0, 3.4 Hz, 4H), 1.84 (ddd, *J* = 14.1, 8.9, 3.6 Hz, 2H), 1.75 (t, *J* = 13.3 Hz, 2H), 1.68 (dd, *J* = 6.9, 2.3 Hz, 2H), 1.63 − 1.60 (m, 2H), 1.61 − 1.58 (m, 2H), 1.57 (d, *J* = 5.3 Hz, 2H), 1.55 (t, *J* = 5.0 Hz, 2H), 1.53 − 1.50 (m, 2H), 1.44 (td, *J* = 13.2, 4.0 Hz, 2H), 1.25 (s, 3H, CH_3_), 1.15 (s, 3H, CH_3_), 1.03 (dt, *J* = 14.2, 3.7 Hz, 2H), 0.98 (s, 3H, CH_3_), 0.89 (d, *J* = 12.7 Hz, 6H, 2CH_3_), 0.84 (s, 3H), 0.77 (s, 3H, CH_3_), 0.70 (s, 3H, CH_3_); ^13^C NMR (151 MHz, Chloroform-*d*) δ 178.61, 145.02(C6-Tacrine), 124.67(C5-Tacrine), 124.59(C7-Tacrine), 122.79(C8-Tacrine), 55.06, 48.76, 47.47, 46.77, 46.32, 42.30, 42.10, 39.35, 38.93, 38.76, 38.45, 36.93, 34.13, 32.95, 32.61, 32.31, 30.72, 29.71, 29.33, 28.95, 28.09, 27.27, 27.22, 27.14, 27.05, 25.71, 24.66, 23.87, 23.56, 23.50, 22.80, 22.34, 18.27, 16.97, 15.57, 15.33, 14.13; ESI-MS *m/z* 714.5 [M + H]^+^.

##### N-(4-((6-chloro-1,2,3,4-tetrahydroacridin-9-yl)amino)butyl)-olean-12-en-28- amide B2

White solid (0.19 g, 0.26 mmol), yield 25.59%. ^1^H NMR (600 MHz, Chloroform-*d*) δ 12.48 (s, 1H, olean amide), 8.16 (d, *J* = 9.3 Hz, 1H, H8-Tacrine), 7.92 (d, *J* = 2.1 Hz, 1H, H5-Tacrine), 7.32 (dd, *J* = 9.2, 2.1 Hz, 1H, H7-Tacrine), 6.31 (t, *J* = 5.9 Hz, 1H, -NH-), 5.41 (t, *J* = 3.6 Hz, 1H, CH = C), 3.96 (q, *J* = 6.5 Hz, 2H), 3.42 (dq, *J* = 13.5, 6.8 Hz, 1H), 3.21 (dd, *J* = 11.5, 4.4 Hz, 1H), 3.15 (dt, *J* = 13.3, 7.0 Hz, 1H), 3.09 (t, *J* = 6.1 Hz, 2H), 2.63 (t, *J* = 6.0 Hz, 2H), 2.58 (dd, *J* = 12.9, 4.4 Hz, 1H), 2.01 (td, *J* = 13.8, 3.7 Hz, 2H), 1.92 − 1.88 (m, 4H), 1.86 (ddd, *J* = 13.7, 8.2, 5.0 Hz, 2H), 1.75 (t, *J* = 13.3 Hz, 2H), 1.72 − 1.64 (m, 6H), 1.64 − 1.54 (m, 6H), 1.53 − 1.49 (m, 2H), 1.25 (q, *J* = 7.6, 5.5 Hz, 4H), 1.22 − 1.18 (m, 2H), 1.16 (s, 3H, CH_3_), 0.98 (s, 3H, CH_3_), 0.90 (s, 6H, 2CH_3_), 0.87 (s, 3H, CH_3_), 0.76 (d, *J* = 10.8 Hz, 6H, 2CH_3_); ^13^C NMR (151 MHz, Chloroform-*d*) δ 179.34, 156.02, 151.21, 144.41(C6-Tacrine), 139.02, 138.83, 126.62(C5-Tacrine), 126.02(C7-Tacrine), 123.14(C8-Tacrine), 118.65, 113.89, 111.73, 55.08, 47.92, 47.51, 46.67, 46.32, 42.02, 41.96, 39.39, 38.76, 38.69, 38.44, 36.96, 34.15, 32.98, 32.84, 32.39, 30.71, 29.71, 29.35, 29.33, 28.43, 28.10, 27.54, 27.30, 27.22, 27.14, 26.84, 25.78, 23.81, 23.56, 23.52, 23.49, 21.74, 20.54, 18.28, 17.04, 15.59, 15.33, 14.13; ESI-MS *m/z* 742.5 [M + H]^+^.

##### N-(5-((6-chloro-1,2,3,4-tetrahydroacridin-9-yl)amino)pentyl)-olean-12-en-28- amide B3

White solid (0.27 g, 0.36 mmol), yield 35.69%. ^1^H NMR (600 MHz, Chloroform-*d*) δ 10.13 (s, 1H, olean amide), 8.30 (d, *J* = 8.8 Hz, 1H, H8-Tacrine), 7.98 (dd, *J* = 7.7, 0.9 Hz, 1H, H5-Tacrine), 7.38 (dd, *J* = 8.7, 7.6 Hz, 1H, H7-Tacrine), 6.86 (t, *J* = 6.2 Hz, 1H, -NH-), 5.37 − 5.33 (m, 1H, CH = C), 4.11 − 4.00 (m, 2H), 3.69 − 3.66 (m, 6H), 3.64 − 3.61 (m, 6H), 3.16 (d, *J* = 4.3 Hz, 4H), 3.12 (d, *J* = 4.3 Hz, 4H), 3.06 (d, *J* = 5.6 Hz, 2H), 2.85 (s, 1H), 2.62 (s, 1H), 1.97 (td, *J* = 13.9, 3.8 Hz, 1H), 1.87 (dq, *J* = 14.0, 6.6 Hz, 4H), 1.77 (dd, *J* = 8.9, 3.6 Hz, 2H), 1.65 (t, *J* = 13.5 Hz, 1H), 1.60 − 1.51 (m, 4H), 1.49 (dd, *J* = 15.4, 4.7 Hz, 4H), 1.06 (s, 3H, CH_3_), 0.88 (s, 3H, CH_3_), 0.83 (d, *J* = 4.4 Hz, 6H, 2CH_3_), 0.67 (d, *J* = 10.0 Hz, 6H, 2CH_3_), 0.39 (s, 3H, CH_3_); ^13^C NMR (151 MHz, Chloroform-*d*) δ 182.22, 165.55, 157.70, 143.32(C6-Tacrine), 136.61(C5-Tacrine), 125.69(C7-Tacrine), 123.47(C8-Tacrine), 112.34, 78.71, 77.37, 77.16, 76.95, 55.60, 55.49, 55.36, 54.98, 52.15, 47.34, 46.37, 46.20, 43.44, 41.71, 41.34, 41.20, 39.62, 39.20, 38.67, 38.63, 38.63, 38.59, 38.56, 38.34, 37.95, 36.82, 33.96, 32.95, 32.77, 32.25, 31.83, 30.60, 29.62, 29.24, 28.74, 28.02, 27.21, 27.01, 25.82, 25.70, 23.45, 23.30, 23.26, 21.71, 20.68, 18.40, 18.27, 18.15, 17.00, 16.89, 16.76, 16.52, 15.60, 15.54, 15.12, 12.71; ESI-MS *m/z* 756.5 [M + H]^+^.

##### N-(6-((6-chloro-1,2,3,4-tetrahydroacridin-9-yl)amino)hexyl)-olean-12-en-28- amide B4

White solid (0.27 g, 0.35 mmol), yield 35.04%. ^1^H NMR (600 MHz, Chloroform-*d*) δ 8.33 (d, *J* = 2.1 Hz, 1H, H5-Tacrine), 8.19 (d, *J* = 9.2 Hz, 1H, H8-Tacrine), 7.34 (dd, *J* = 9.2, 2.2 Hz, 1H, H7-Tacrine), 6.08 (t, *J* = 5.9 Hz, 1H, -NH-), 5.36 (t, *J* = 3.7 Hz, 1H, CH = C), 3.87 (t, *J* = 7.0 Hz, 2H), 3.40 (dd, *J* = 13.5, 6.7 Hz, 1H), 3.22 (dd, *J* = 8.2, 4.5 Hz, 2H), 3.19 (d, *J* = 4.6 Hz, 1H), 3.06 − 3.01 (m, 1H), 2.66 − 2.62 (m, 2H), 2.53 − 2.48 (m, 1H), 1.97 (td, *J* = 13.8, 3.9 Hz, 2H), 1.92 (d, *J* = 5.9 Hz, 2H), 1.89 (dd, *J* = 8.8, 3.9 Hz, 4H), 1.75 (t, *J* = 13.4 Hz, 2H), 1.71 − 1.64 (m, 2H), 1.60 (dd, *J* = 6.9, 2.6 Hz, 4H), 1.58 − 1.53 (m, 4H), 1.51 (dd, *J* = 8.5, 4.3 Hz, 2H), 1.47 (dd, *J* = 8.2, 6.6 Hz, 2H), 1.45 (d, *J* = 3.8 Hz, 1H), 1.37 − 1.32 (m, 2H), 1.28 − 1.21 (m, 4H), 1.18 (d, *J* = 6.7 Hz, 2H), 1.15 (s, 3H, CH_3_), 1.03 (d, *J* = 13.7 Hz, 2H), 0.98 (s, 3H, CH_3_), 0.89 (d, *J* = 5.5 Hz, 6H, 2CH_3_), 0.87 (s, 3H, CH_3_), 0.75 (d, *J* = 12.8 Hz, 6H, 2CH_3_); ^13^C NMR (151 MHz, Chloroform-*d*) δ 178.78, 155.25, 144.85(C6-Tacrine), 126.10(C5-Tacrine), 125.87(C7-Tacrine), 122.84(C8-Tacrine), 114.47, 111.65, 55.04, 50.84, 48.69, 47.47, 46.75, 46.32, 45.91, 42.18, 42.06, 39.35, 38.75, 38.43, 36.94, 34.12, 32.95, 32.70, 32.33, 30.71, 30.23, 29.69, 29.42, 28.08, 27.27, 27.13, 25.73, 24.08, 23.85, 23.75, 23.58, 23.50, 21.92, 20.72, 18.26, 16.98, 15.58, 15.35, 14.12, 8.65; ESI-MS *m/z* 770.5 [M + H]^+^; HRMS(ESI) *m/z* [M + H]+ calcd for C_49_H_73_N_3_ClO_2_^+^ 770.5391, found 770.5406.

##### N-(2-((5-chloro-1,2,3,4-tetrahydroacridin-9-yl)amino)ethyl)-olean-12-en-28- amide C1

Yellow solid (0.33 g, 0.46 mmol), yield 46.18%. ^1^H NMR (600 MHz, Chloroform-*d*) δ 8.69 (dd, *J* = 4.4, 1.4 Hz, 1H, H7-Tacrine), 8.38 (dd, *J* = 8.3, 1.4 Hz, H6-Tacrine, 1H), 7.40 (d, *J* = 4.4 Hz, 1H, H8-Tacrine), 5.34 (t, *J* = 3.7 Hz, 1H, CH = C), 3.22 (dd, *J* = 11.4, 4.2 Hz, 1H), 3.11 (s, 1H), 2.98 − 2.91 (m, 1H), 2.28 (td, *J* = 13.9, 3.8 Hz, 1H), 2.18 (td, *J* = 13.7, 4.5 Hz, 1H), 2.13 − 2.05 (m, 2H), 2.04 − 1.98 (m, 1H), 1.87 (dt, *J* = 11.1, 4.4 Hz, 2H), 1.76 (t, *J* = 13.7 Hz, 1H), 1.61 (dd, *J* = 11.9, 3.1 Hz, 4H), 1.57 − 1.54 (m, 2H), 1.54 − 1.47 (m, 2H), 1.45 − 1.42 (m, 2H), 1.42 − 1.39 (m, 1H), 1.35 (d, *J* = 14.3 Hz, 1H), 1.27 (s, 1H), 1.25 (s, 2H), 1.22 (s, 3H, CH_3_), 1.00 (s, 3H, CH_3_), 0.98 (s, 3H, CH_3_), 0.96 (s, 3H, CH_3_), 0.90 (s, 3H, CH_3_), 0.87 (s, 1H), 0.85 (s, 3H, CH_3_), 0.79 (s, 3H, CH_3_), 0.75 (dd, *J* = 11.7, 1.9 Hz, 1H); ^13^C NMR (151 MHz, Chloroform-*d*) δ 173.29, 151.48(C5-Tacrine), 142.07, 140.79, 134.95(C6-Tacrine), 129.17(C7-Tacrine), 123.66(C8-Tacrine), 120.56, 55.20, 47.56, 47.44, 45.54, 41.87, 41.59, 39.43, 38.70, 38.47, 36.95, 33.64, 32.88, 32.73, 32.11, 30.56, 29.62, 28.05, 28.03, 27.12, 25.66, 23.46, 23.40, 23.25, 18.29, 16.87, 15.51, 15.33; ESI-MS *m/z* 714.5 [M + H]^+^.

##### N-(3-((5-chloro-1,2,3,4-tetrahydroacridin-9-yl)amino)propyl)-olean-12-en- 28-amide C2

Yellow solid (0.17 g, 0.23 mmol), yield 23.34%. ^1^H NMR (600 MHz, Chloroform-*d*) δ 8.69 (dd, *J* = 4.5, 1.4 Hz, 1H, H6-Tacrine), 8.38 (dd, *J* = 8.4, 1.4 Hz, 1H, H8-Tacrine), 7.40 (dd, *J* = 8.4, 4.5 Hz, 1H, H7-Tacrine), 5.34 (t, *J* = 3.7 Hz, 1H, CH = C), 3.22 (dd, *J* = 11.3, 4.3 Hz, 1H), 2.97 − 2.91 (m, 1H), 2.28 (td, *J* = 13.9, 3.8 Hz, 1H), 2.18 (td, *J* = 13.8, 4.5 Hz, 1H), 2.13 − 2.06 (m, 2H), 2.01 (dq, *J* = 13.9, 2.1 Hz, 1H), 1.87 (dt, *J* = 11.1, 4.5 Hz, 2H), 1.76 (t, *J* = 13.7 Hz, 1H), 1.63 − 1.58 (m, 4H), 1.58 − 1.54 (m, 2H), 1.50 (dt, *J* = 13.7, 4.3 Hz, 2H), 1.47 − 1.45 (m, 1H), 1.43 (dd, *J* = 7.3, 2.4 Hz, 1H), 1.41 (q, *J* = 2.6 Hz, 2H), 1.35 (dt, *J* = 14.3, 3.5 Hz, 1H), 1.28 − 1.26 (m, 1H), 1.25 (s, 3H, CH_3_), 1.22 (s, 3H, CH_3_), 1.10 (s, 1H), 1.00 (s, 3H, CH_3_), 0.98 (s, 3H, CH_3_), 0.96 (s, 3H, CH_3_), 0.90 (s, 3H, CH_3_), 0.85 (s, 3H), 0.79 (s, 3H, CH_3_), 0.75 (dd, *J* = 11.7, 2.0 Hz, 1H); ^13^C NMR (151 MHz, Chloroform-*d*) δ 172.34, 150.54(C5-Tacrine), 141.13, 139.84, 134.01, 128.23(C6-Tacrine), 122.72(C7-Tacrine), 119.62(C8-Tacrine), 54.26, 46.62, 46.50, 44.60, 40.93, 40.65, 38.49, 37.76, 37.53, 37.13, 36.01, 32.70, 31.94, 31.79, 31.17, 30.22, 29.62, 28.68, 27.11, 27.09, 26.18, 24.72, 22.52, 22.46, 22.31, 17.35, 15.93, 14.57, 14.39; ESI-MS *m/z* 728.4 [M + H]^+^.

##### N-(4-((5-chloro-1,2,3,4-tetrahydroacridin-9-yl)amino)butyl)-olean-12-en-28- amide C3

Yellow solid (0.25 g, 0.34 mmol), yield 33.67%. ^1^H NMR (600 MHz, Chloroform-*d*) δ 8.17 (d, *J* = 8.7 Hz, 1H, H6-Tacrine), 7.76 (d, *J* = 7.6 Hz, 1H, H8-Tacrine), 7.41 (t, *J* = 8.1 Hz, 1H, H7-Tacrine), 6.22 (t, *J* = 5.9 Hz, 1H, -NH-), 5.37 (d, *J* = 3.7 Hz, 1H, CH = C), 3.88 (t, *J* = 7.2 Hz, 2H), 3.37 (dd, *J* = 13.5, 6.8 Hz, 1H), 3.19 (dd, *J* = 11.6, 4.2 Hz, 1H), 3.13 − 3.09 (m, 2H), 3.07 (d, *J* = 7.1 Hz, 1H), 2.69 (s, 2H), 2.59 − 2.53 (m, 1H), 1.98 (td, *J* = 13.9, 3.9 Hz, 2H), 1.91 (s, 4H), 1.89 − 1.85 (m, 2H), 1.82 (t, *J* = 7.2 Hz, 2H), 1.73 (t, *J* = 13.4 Hz, 2H), 1.64 (dd, *J* = 13.2, 6.2 Hz, 4H), 1.55 (d, *J* = 6.3 Hz, 2H), 1.52 (d, *J* = 9.1 Hz, 4H), 1.43 (td, *J* = 12.8, 4.1 Hz, 2H), 1.24 (s, 3H), 1.14 (s, 3H, CH_3_), 1.08 − 1.00 (m, 2H), 0.96 (s, 3H, CH_3_), 0.88 (s, 6H, 2CH_3_), 0.84 (s, 3H, CH_3_), 0.75 (s, 3H, CH_3_), 0.71 (s, 3H, CH_3_); ^13^C NMR (151 MHz, Chloroform-*d*) δ 177.81, 154.97, 151.51, 143.46(C5-Tacrine), 135.24, 131.02(C6-Tacrine), 123.99(C7-Tacrine), 123.85, 123.09, 121.96(C8-Tacrine), 116.65, 112.64, 54.06, 52.43, 47.44, 46.49, 45.65, 45.24, 40.96, 40.90, 38.34, 37.75, 37.73, 37.42, 35.93, 33.15, 31.99, 31.76, 31.39, 29.69, 28.89, 28.68, 27.08, 26.71, 26.28, 26.12, 25.78, 24.75, 22.70, 22.57, 22.45, 20.84, 19.95, 17.26, 16.00, 14.58, 14.29; ESI-MS *m/z* 742.5 [M + H]^+^.

##### N-(5-((5-chloro-1,2,3,4-tetrahydroacridin-9-yl)amino)pentyl)-olean-12-en- 28-amide C4

Yellow solid (0.23 g, 0.30 mmol), yield 30.40%. ^1^H NMR (600 MHz, Chloroform-*d*) δ 8.16 (d, *J* = 8.7 Hz, 1H, H6-Tacrine), 7.76 (d, *J* = 7.5 Hz, 1H, H8-Tacrine), 7.40 (t, *J* = 8.2 Hz, 1H, H7-Tacrine), 6.06 (t, *J* = 5.9 Hz, 1H, -NH-), 5.36 (d, *J* = 3.7 Hz, 1H, CH = C), 3.80 (t, *J* = 7.2 Hz, 2H), 3.73 (d, *J* = 6.3 Hz, 1H), 3.35 (dq, *J* = 13.8, 7.1 Hz, 1H), 3.20 (dd, *J* = 11.5, 4.2 Hz, 1H), 3.11 (d, *J* = 5.4 Hz, 2H), 3.02 (dq, *J* = 12.8, 6.4 Hz, 1H), 2.68 (d, *J* = 5.9 Hz, 2H), 2.53 (dd, *J* = 13.0, 4.3 Hz, 1H), 1.94 − 1.90 (m, 4H), 1.88 (d, *J* = 8.0 Hz, 2H), 1.85 − 1.83 (m, 2H), 1.81 (d, *J* = 6.7 Hz, 1H), 1.74 (t, *J* = 13.4 Hz, 2H), 1.67 − 1.61 (m, 2H), 1.59 (d, *J* = 5.8 Hz, 2H), 1.56 (s, 2H), 1.53 (d, *J* = 7.3 Hz, 3H), 1.51 (s, 2H), 1.42 (t, *J* = 7.6 Hz, 4H), 1.20 (d, *J* = 26.7 Hz, 3H), 1.14 (s, 3H, CH_3_), 1.05 − 0.99 (m, 2H), 0.97 (s, 3H, CH_3_), 0.89 (s, 6H, 2CH_3_), 0.86 (s, 3H, CH_3_), 0.76 (s, 3H, CH_3_), 0.73 (s, 3H, CH_3_); ^13^C NMR (151 MHz, Chloroform-*d*) δ 177.49, 154.42, 152.25, 143.72(C5-Tacrine), 136.11, 130.63, 124.88(C6-Tacrine), 123.62, 122.96(C7-Tacrine), 121.85(C8-Tacrine), 117.09, 112.95, 97.44, 66.96, 66.41, 54.06, 48.05, 46.50, 45.72, 45.25, 41.09, 41.02, 38.34, 38.01, 37.73, 37.43, 35.93, 33.15, 32.28, 31.98, 31.68, 31.37, 30.91, 29.70, 29.45, 29.18, 28.68, 28.64, 28.35, 28.17, 27.40, 27.08, 26.28, 26.13, 24.73, 24.60, 23.10, 22.77, 22.70, 22.58, 22.48, 22.37, 21.68, 20.94, 20.13, 17.26, 15.97, 14.57, 14.32, 13.11; ESI-MS *m/z* 756.5 [M + H]^+^.

##### N-(6-((5-chloro-1,2,3,4-tetrahydroacridin-9-yl)amino)hexyl)-olean-12-en-28- amide C5

Yellow solid (0.17 g, 0.22 mmol), yield 22.06%. ^1^H NMR (600 MHz, Chloroform-*d*) δ 8.69 (d, *J* = 4.4 Hz, 1H, H6-Tacrine), 8.38 (dt, *J* = 8.4, 1.6 Hz, 1H, H8-Tacrine), 7.40 (ddd, *J* = 8.4, 4.5, 1.7 Hz, 1H, H7-Tacrine), 7.32 − 7.31 (m, 1H, -NH-), 5.34 (t, *J* = 4.1 Hz, 1H, CH = C), 3.78 (d, *J* = 10.7 Hz, 1H), 3.52 (s, 1H), 3.27 − 3.16 (m, 2H), 2.94 (dd, *J* = 13.9, 4.8 Hz, 1H), 2.52 − 2.45 (m, 1H), 2.35 − 2.24 (m, 2H), 2.23 − 2.15 (m, 2H), 2.10 (td, *J* = 11.5, 10.4, 3.7 Hz, 3H), 2.05 − 1.98 (m, 2H), 1.95 − 1.88 (m, 2H), 1.88 − 1.86 (m, 2H), 1.76 (td, *J* = 13.0, 12.3, 4.8 Hz, 4H), 1.63 − 1.58 (m, 4H), 1.57 − 1.53 (m, 4H), 1.43 (ddt, *J* = 13.2, 9.5, 3.5 Hz, 4H), 1.40 − 1.31 (m, 4H), 1.30 − 1.23 (m, 4H), 1.22 (s, 3H, CH_3_), 1.17 − 1.13 (m, 2H), 1.00 (s, 3H, CH_3_), 0.98 (s, 3H, CH_3_), 0.96 (s, 3H, CH_3_), 0.89 (s, 3H, CH_3_), 0.85 (s, 3H, CH_3_), 0.78 (s, 3H, CH_3_); ^13^C NMR (151 MHz, Chloroform-*d*) δ 177.39, 173.35, 151.55(C5-Tacrine), 144.63, 142.13, 140.84, 135.01, 129.33(C6-Tacrine), 129.23, 128.29, 123.72(C7-Tacrine), 122.60, 120.63(C8-Tacrine), 55.25, 55.09, 47.62, 47.52, 47.50, 46.75, 46.30, 45.60, 42.28, 42.17, 41.92, 41.64, 39.48, 39.37, 38.76, 38.73, 38.60, 38.52, 38.47, 37.01, 36.96, 34.13, 33.70, 32.96, 32.94, 32.78, 32.53, 32.16, 30.69, 30.62, 28.11, 28.09, 27.31, 27.18, 27.15, 25.72, 25.59, 23.60, 23.52, 23.50, 23.46, 23.31, 18.35, 18.25, 17.56, 16.92, 15.58, 15.56, 15.38; ESI-MS *m/z* 770.5 [M + H]^+^.

##### N-(2-((6-bromo-1,2,3,4-tetrahydroacridin-9-yl)amino)ethyl)-olean-12-en-28- amide D1

Brown solid (0.15 g, 0.20 mmol), yield 19.78%. ^1^H NMR (600 MHz, Chloroform-*d*) δ 8.29 (s, 1H, H5-Tacrine), 8.08 (d, *J* = 9.4 Hz, 1H, H7-Tacrine), 7.30 (d, *J* = 9.3 Hz, 1H, H8-Tacrine), 5.43 (s, 1H, CH = C), 4.15 (s, 2H), 3.84 (s, 2H), 3.66 (s, 2H), 3.23 − 3.09 (m, 4H), 2.72 − 2.57 (m, 4H), 2.03 (dd, *J* = 13.7, 3.9 Hz, 2H), 1.91 − 1.86 (m, 4H), 1.77 − 1.68 (m, 4H), 1.64 − 1.57 (m, 4H), 1.57 − 1.51 (m, 4H), 1.29 − 1.23 (m, 4H), 1.15 (s, 3H, CH_3_), 0.97 (s, 3H, CH_3_), 0.91 (d, *J* = 4.7 Hz, 6H, 2CH_3_), 0.83 (s, 3H, CH_3_), 0.75 (s, 3H, CH_3_), 0.70 (d, *J* = 11.4 Hz, 2H), 0.64 (s, 3H, CH_3_); ^13^C NMR (151 MHz, Chloroform-*d*) δ 180.97, 143.22(C6-Tacrine), 126.80(C5-Tacrine), 125.38(C7-Tacrine), 122.34(C8-Tacrine), 54.04, 52.42, 50.64, 46.43, 45.55, 45.35, 40.94, 40.79, 38.95, 38.34, 37.73, 37.40, 35.93, 33.04, 31.95, 31.78, 31.22, 29.72, 28.77, 28.69, 28.35, 28.31, 27.63, 27.05, 26.28, 26.20, 26.11, 24.76, 22.97, 22.66, 22.60, 22.47, 21.68, 21.05, 19.78, 17.22, 15.76, 14.54, 14.34, 14.30, 13.12; ESI-MS *m/z* 758.5 [M + H]^+^.

##### N-(3-((6-romo-1,2,3,4-tetrahydroacridin-9-yl)amino)propyl)-olean-12-en- 28-amide D2

Brown solid (0.18 g, 0.23 mmol), yield 23.30%. ^1^H NMR (600 MHz, DMSO-*d*_6_) δ 12.57 (s, 1H, olean amide), 7.57 (d, *J* = 8.8 Hz, 1H, H5-Tacrine), 7.51 (s, 1H), 7.14 (t, *J* = 9.5 Hz, 1H, H7-Tacrine), 7.09 (d, *J* = 9.3 Hz, 1H, H8-Tacrine), 4.41 (t, *J* = 3.7 Hz, 1H, CH = C), 3.59 (d, *J* = 5.1 Hz, 1H), 3.32 (s, 1H), 3.25 (s, 1H), 2.93 (td, *J* = 6.6, 3.9 Hz, 1H), 2.73 (s, 1H), 2.45 (dd, *J* = 7.4, 4.2 Hz, 1H), 2.32 (s, 1H), 2.25 (d, *J* = 5.5 Hz, 1H), 1.21 (dd, *J* = 14.0, 3.9 Hz, 2H), 1.14 (s, 4H), 1.06 − 0.98 (m, 2H), 0.91 (t, *J* = 13.6 Hz, 2H), 0.85 − 0.79 (m, 2H), 0.70 (d, *J* = 7.8 Hz, 3H), 0.68 − 0.64 (m, 2H), 0.62 (d, *J* = 5.2 Hz, 2H), 0.60 (s, 1H), 0.58 (s, 2H), 0.57 (d, *J* = 2.1 Hz, 4H), 0.55 (d, *J* = 1.0 Hz, 3H, CH_3_), 0.54 (s, 1H), 0.38 − 0.35 (m, 1H), 0.31 (s, 3H, CH_3_), 0.26 (d, *J* = 13.7 Hz, 1H), 0.17 (s, 3H, CH_3_), 0.16 (s, 3H, CH_3_), 0.13 (s, 3H, CH_3_), 0.08 (t, *J* = 12.5 Hz, 2H), −0.06 (s, 3H, CH_3_), −0.13 (s, 3H, CH_3_); ^13^C NMR (151 MHz, Chloroform-*d*) δ 181.33, 159.18, 114.35, 92.19, 91.24, 76.16, 75.96, 75.88, 75.52, 74.04, 71.09, 70.42, 70.30, 69.60, 68.00, 65.80, 64.52, 64.42, 63.12, 61.03, 60.40, 59.86, 58.94, 58.16, 55.72, 55.40, 54.37, 53.83, 53.54, 52.35, 50.13; ESI-MS *m/z* 772.4 [M + H]^+^.

##### N-(4-((6-bromo-1,2,3,4-tetrahydroacridin-9-yl)amino)butyl)-olean-12-en-28- amide D3

Brown solid (0.22 g, 0.28 mmol), yield 27.98%. ^1^H NMR (600 MHz, Chloroform-*d*) δ 8.18 (d, *J* = 9.3 Hz, 1H, H8-Tacrine), 7.65 (d, *J* = 2.2 Hz, 1H, H5-Tacrine), 7.21 (dd, *J* = 9.3, 2.1 Hz, 1H, H7-Tacrine), 6.83 (t, *J* = 6.2 Hz, 1H, -NH-), 5.45 (t, *J* = 3.7 Hz, 1H, CH = C), 4.12 − 3.99 (m, 2H), 3.79 (ddd, *J* = 12.7, 7.3, 2.6 Hz, 1H), 3.63 − 3.56 (m, 1H), 3.19 (dd, *J* = 11.5, 4.3 Hz, 1H), 3.01 (d, *J* = 6.1 Hz, 2H), 2.65 (d, *J* = 7.8 Hz, 3H), 2.04 (td, *J* = 13.7, 3.9 Hz, 1H), 1.93 (d, *J* = 5.8 Hz, 2H), 1.90 − 1.86 (m, 3H), 1.74 (t, *J* = 13.5 Hz, 1H), 1.70 − 1.62 (m, 2H), 1.61 − 1.55 (m, 3H), 1.51 (dd, *J* = 10.5, 7.0 Hz, 2H), 1.45 − 1.39 (m, 2H), 1.39 − 1.32 (m, 2H), 1.27 − 1.22 (m, 2H), 1.22 − 1.17 (m, 2H), 1.14 (s, 3H, CH_3_), 1.04 (dd, *J* = 13.7, 3.1 Hz, 1H), 0.95 (s, 3H, CH_3_), 0.90 (d, *J* = 5.9 Hz, 6H, 2CH_3_), 0.81 (s, 3H, CH_3_), 0.73 (s, 3H, CH_3_), 0.68 (dd, *J* = 11.9, 1.9 Hz, 1H), 0.60 (s, 3H, CH_3_); ^13^C NMR (151 MHz, Chloroform-*d*) δ 181.29, 181.27, 164.75, 154.97, 154.94, 149.85, 142.58(C6-Tacrine), 142.56, 138.50, 137.53, 137.49, 126.19(C5-Tacrine), 124.39, 124.34(C7-Tacrine), 122.67(C8-Tacrine), 117.81, 113.10, 111.32, 111.28, 54.04, 50.79, 46.44, 45.48, 45.32, 40.85, 40.60, 40.58, 38.65, 38.32, 37.70, 37.66, 37.39, 35.89, 33.06, 31.96, 31.86, 31.26, 29.67, 28.68, 27.72, 27.05, 26.27, 26.10, 24.75, 22.66, 22.52, 22.48, 22.41, 20.92, 19.84, 17.21, 15.64, 14.54, 14.20; ESI-MS *m/z* 786.4 [M + H]^+^.

##### N-(5-((6-bromo-1,2,3,4-tetrahydroacridin-9-yl)amino)pentyl)-olean-12-en- 28-amide D4

Brown solid (0.33 g, 0.41 mmol), yield 41.23%. ^1^H NMR (600 MHz, Chloroform-*d*) δ 8.02 (d, *J* = 9.3 Hz, 1H, H8-Tacrine), 7.94 (d, *J* = 2.1 Hz, 1H, H5-Tacrine), 7.44 (dd, *J* = 8.9, 2.1 Hz, 1H, H7-Tacrine), 6.17 (t, *J* = 5.8 Hz, 1H, -NH-), 5.36 (d, *J* = 3.6 Hz, 1H, CH = C), 3.78 (d, *J* = 7.8 Hz, 2H), 3.36 (dd, *J* = 13.6, 6.8 Hz, 1H), 3.19 (dd, *J* = 11.4, 4.6 Hz, 1H), 3.09 − 3.02 (m, 1H), 2.98 (d, *J* = 6.4 Hz, 2H), 2.60 (d, *J* = 6.3 Hz, 2H), 2.57 − 2.50 (m, 1H), 2.00 − 1.94 (m, 1H), 1.90 (s, 2H), 1.86 (t, *J* = 4.9 Hz, 4H), 1.82 (d, *J* = 7.0 Hz, 2H), 1.73 (t, *J* = 13.3 Hz, 1H), 1.68 − 1.60 (m, 2H), 1.57 (d, *J* = 5.3 Hz, 2H), 1.55 (d, *J* = 6.8 Hz, 4H), 1.50 (d, *J* = 12.8 Hz, 2H), 1.46 − 1.39 (m, 3H), 1.36 − 1.29 (m, 2H), 1.23 (d, *J* = 15.9 Hz, 2H), 1.13 (s, 3H, CH_3_), 1.02 (d, *J* = 13.5 Hz, 1H), 0.96 (s, 3H, CH_3_), 0.88 (s, 6H, 2CH_3_), 0.83 (s, 3H, CH_3_), 0.74 (d, *J* = 7.0 Hz, 6H, 2CH_3_), 0.69 (d, *J* = 11.8 Hz, 1H); ^13^C NMR (151 MHz, Chloroform-*d*) δ 177.39, 173.35, 151.55(C6-Tacrine), 142.13, 140.84, 135.01, 129.33(C5-Tacrine), 129.23, 128.29, 123.72(C7-Tacrine), 122.60, 120.63(C8-Tacrine), 55.25, 55.09, 47.62, 47.52, 47.50, 46.75, 46.30, 45.60, 42.28, 42.17, 41.93, 41.64, 39.48, 39.37, 38.77, 38.76, 38.73, 38.60, 38.53, 38.47, 37.01, 36.96, 34.13, 33.70, 32.96, 32.94, 32.78, 32.53, 32.17, 30.69, 30.62, 28.11, 28.09, 27.31, 27.18, 27.15, 25.72, 25.59, 23.60, 23.52, 23.50, 23.46, 23.31, 18.35, 18.25, 17.56, 16.92, 15.58, 15.56, 15.39; ESI-MS *m/z* 800.5 [M + H]^+^; HRMS(ESI) *m/z* [M + H]+ calcd for C_48_H_71_N_3_BrO_2_^+^ 800.4730, found 800.4744.

##### N-(6-((6-bromo-1,2,3,4-tetrahydroacridin-9-yl)amino)hexyl)-olean-12-en-28- amide D5

Brown solid (0.21 g, 0.26 mmol), yield 25.79%. ^1^H NMR (600 MHz, Chloroform-*d*) δ 8.07 (d, *J* = 2.0 Hz, 1H, H5-Tacrine), 8.05 (d, *J* = 9.2 Hz, 1H, H8-Tacrine), 7.50 (dd, *J* = 9.1, 1.9 Hz, 1H, H7-Tacrine), 6.01 (t, *J* = 5.5 Hz, 1H, -NH-), 5.37 (d, *J* = 3.7 Hz, 1H, CH = C), 3.85 (t, *J* = 7.3 Hz, 2H), 3.35 − 3.29 (m, 1H), 3.21 (dd, *J* = 11.5, 4.4 Hz, 1H), 3.04 (t, *J* = 6.2 Hz, 2H), 3.02 − 2.95 (m, 1H), 2.61 (t, *J* = 6.2 Hz, 2H), 2.50 (dd, *J* = 12.9, 4.4 Hz, 1H), 1.99 − 1.93 (m, 3H), 1.91 (dd, *J* = 8.9, 3.6 Hz, 4H), 1.81 (t, *J* = 7.5 Hz, 2H), 1.75 − 1.67 (m, 2H), 1.60 (dt, *J* = 11.7, 3.7 Hz, 4H), 1.57 − 1.52 (m, 4H), 1.46 (s, 3H), 1.43 − 1.38 (m, 3H), 1.38 − 1.33 (m, 2H), 1.31 (d, *J* = 8.8 Hz, 3H), 1.16 (s, 3H, CH_3_), 1.06 1.02 (m, 1H), 0.98 (s, 3H, CH_3_), 0.90 (s, 9H, 3CH_3_), 0.77 (d, *J* = 5.0 Hz, 6H, 2CH_3_), 0.72 (d, *J* = 11.7 Hz, 1H); ^13^C NMR (151 MHz, Chloroform-*d*) δ 177.40, 154.33, 151.25, 143.96(C6-Tacrine), 139.48, 127.40(C5-Tacrine), 125.86, 125.29(C7-Tacrine), 122.38, 121.81(C8-Tacrine), 113.82, 111.32, 54.06, 52.42, 49.83, 47.87, 46.50, 45.75, 45.26, 41.35, 41.10, 38.55, 38.36, 37.75, 37.46, 35.95, 33.13, 31.96, 31.57, 31.37, 29.72, 28.69, 28.64, 28.51, 28.47, 28.41, 28.35, 28.33, 28.31, 28.24, 28.14, 28.11, 28.03, 27.09, 26.28, 26.11, 26.04, 25.55, 24.71, 22.83, 22.56, 22.53, 22.28, 20.83, 19.88, 17.27, 15.96, 14.59, 14.37, 13.11; ESI-MS *m/z* 814.4 [M + H]^+^.

##### N-(2-((5-bromo-1,2,3,4-tetrahydroacridin-9-yl)amino)ethyl)-olean-12-en-28- amide E1

Brown solid (0.16 g, 0.21 mmol), yield 21.10%. ^1^H NMR (600 MHz, Chloroform-*d*) δ 8.23 (s, 1H, H8-Tacrine), 8.02 (d, *J* = 9.4 Hz, 1H, H6-Tacrine), 7.24 (d, *J* = 9.3 Hz, 1H, H7-Tacrine), 7.13 (s, 2H), 5.37 (s, 1H, CH = C), 4.08 (s, 2H), 3.78 (s, 1H), 3.59 (s, 1H), 3.15 − 3.09 (m, 2H), 2.63 − 2.57 (m, 2H), 1.88 − 1.76 (m, 6H), 1.56 − 1.44 (m, 6H), 1.42 − 1.34 (m, 3H), 1.33 − 1.25 (m, 3H), 1.21 − 1.17 (m, 2H), 1.15 (d, *J* = 3.6 Hz, 2H), 1.09 (s, 3H, CH_3_), 0.99 (d, *J* = 13.9 Hz, 2H), 0.90 (s, 3H, CH_3_), 0.84 (d, *J* = 4.7 Hz, 6H, 2CH_3_), 0.77 (s, 3H, CH_3_), 0.69 (s, 3H, CH_3_), 0.63 (d, *J* = 11.4 Hz, 2H), 0.58 (s, 3H, CH_3_); ^13^C NMR (151 MHz, Chloroform-*d*) δ 181.92, 155.49, 144.16(C5-Tacrine), 127.74(C6-Tacrine), 126.32(C7-Tacrine), 123.27(C8- Tacrine), 114.25, 111.82, 54.97, 53.36, 51.57, 50.80, 47.37, 46.48, 46.28, 41.87, 41.72, 39.89, 39.27, 39.15, 38.66, 38.33, 36.86, 33.98, 32.89, 32.71, 32.15, 31.85, 30.66, 29.70, 29.63, 29.29, 29.25, 29.18, 28.56, 27.99, 27.22, 27.14, 27.04, 25.69, 23.90, 23.59, 23.53, 23.48, 23.40, 22.62, 21.98, 20.71, 18.16, 16.70, 15.48, 15.24, 14.05; ESI-MS *m/z* 758.4 [M + H]^+^.

##### N-(3-((5-romo-1,2,3,4-tetrahydroacridin-9-yl)amino)propyl)-olean-12-en- 28-amide E2

Brown solid (0.18 g, 0.23 mmol), yield 23.31%. ^1^H NMR (600 MHz, Chloroform-*d*) δ 10.08 (s, 1H, olean amide), 8.30 (d, *J* = 8.6 Hz, 1H, H6-Tacrine), 8.01 (d, *J* = 7.6 Hz, 1H, H8-Tacrine), 7.46 (t, *J* = 8.1 Hz, 1H, H7-Tacrine), 5.47 (d, *J* = 3.5 Hz, 1H, CH = C), 3.64 (td, *J* = 6.7, 4.2 Hz, 4H), 3.23 (dd, *J* = 11.2, 4.8 Hz, 1H), 3.11 (dd, *J* = 7.4, 4.2 Hz, 6H), 2.78 − 2.71 (m, 2H), 2.65 − 2.59 (m, 1H), 2.08 − 2.03 (m, 2H), 1.96 (s, 4H), 1.90 (q, *J* = 3.5 Hz, 2H), 1.74 (t, *J* = 13.4 Hz, 2H), 1.70 − 1.64 (m, 2H), 1.61 − 1.50 (m, 6H), 1.22 (dt, *J* = 21.7, 5.5 Hz, 4H), 1.15 (s, 3H, CH_3_), 1.07 (d, *J* = 6.3 Hz, 2H), 0.97 (s, 3H, CH_3_), 0.90 (s, 6H, 2CH_3_), 0.86 (s, 3H, CH_3_), 0.76 (s, 3H, CH_3_), 0.71 (s, 3H, CH_3_); ^13^C NMR (151 MHz, Chloroform-*d*) δ 177.62, 166.77, 164.96, 156.32, 149.47, 143.50(C5-Tacrine), 135.58, 134.22, 133.22, 131.42, 129.89, 128.90(C6-Tacrine), 128.87, 128.47(C7-Tacrine), 127.78(C8-Tacrine), 124.84, 124.57, 121.96, 115.66, 111.95, 111.74, 77.90, 76.23, 76.02, 75.81, 67.14, 66.75, 54.40, 54.06, 47.97, 46.51, 45.68, 45.23, 40.97, 38.33, 37.98, 37.88, 37.73, 37.71, 37.41, 35.93, 33.15, 31.99, 31.71, 31.37, 30.91, 29.69, 29.55, 29.34, 29.29, 28.68, 28.67, 28.64, 28.60, 28.35, 28.31, 28.08, 28.03, 27.97, 27.91, 27.08, 26.27, 26.20, 26.13, 24.75, 22.96, 22.95, 22.72, 22.69, 22.59, 22.46, 21.97, 21.95, 21.68, 20.52, 19.51, 17.54, 17.26, 16.11, 15.97, 14.57, 14.32, 13.11, 13.04, 10.08, 9.95; ESI-MS *m/z* 772.4 [M + H]^+^.

##### N-(4-((5-bromo-1,2,3,4-tetrahydroacridin-9-yl)amino)butyl)-olean-12-en-28- amide E3

Brown solid (0.25 g, 0.32 mmol), yield 31.79%. ^1^H NMR (600 MHz, Chloroform-*d*) δ 9.92 (s, 1H, olean amide), 8.36 (d, *J* = 8.7 Hz, 1H, H6-Tacrine), 8.06 (d, *J* = 7.6 Hz, 1H, H8-Tacrine), 7.50 (t, *J* = 8.1 Hz, 1H, H7-Tacrine), 6.42 (s, 1H, -NH-), 5.48 (s, 1H, CH = C), 4.05 (d, *J* = 6.6 Hz, 2H), 3.78 − 3.71 (m, 1H), 3.44 (dd, *J* = 13.5, 6.7 Hz, 1H), 3.26 (dd, *J* = 11.5, 4.3 Hz, 1H), 3.14 (s, 2H), 3.01 (s, 1H), 2.76 (s, 2H), 2.68 − 2.63 (m, 1H), 2.00 (s, 6H), 1.97 − 1.92 (m, 4H), 1.79 (t, *J* = 13.4 Hz, 2H), 1.67 (d, *J* = 17.2 Hz, 4H), 1.51 − 1.48 (m, 4H), 1.46 (d, *J* = 6.6 Hz, 3H), 1.31 (s, 6H), 1.20 (s, 3H, CH_3_), 1.10 (d, *J* = 13.3 Hz, 2H), 1.03 (s, 3H, CH_3_), 0.95 (d, *J* = 3.0 Hz, 6H, 2CH_3_), 0.91 (s, 3H, CH_3_), 0.82 (s, 3H, CH_3_), 0.78 (s, 3H, CH_3_); ^13^C NMR (151 MHz, Chloroform-*d*) δ 178.61, 167.70, 165.90, 157.30, 144.55(C5-Tacrine), 136.48, 135.17, 134.17, 132.37(C6-Tacrine), 130.82, 129.42, 128.73(C7-Tacrine), 125.69(C8-Tacrine), 125.50, 122.94, 116.68, 112.97, 112.79, 78.86, 77.16, 76.95, 76.74, 68.09, 67.70, 55.01, 48.30, 47.46, 46.65, 46.19, 42.01, 41.96, 39.28, 38.83, 38.81, 38.68, 38.65, 38.37, 36.89, 34.08, 32.91, 32.57, 32.30, 31.86, 30.64, 30.50, 30.28, 29.97, 29.63, 29.25, 29.12, 29.10, 28.91, 28.85, 28.02, 27.20, 27.08, 25.73, 25.69, 25.40, 23.91, 23.70, 23.67, 23.53, 23.45, 22.92, 22.90, 22.62, 21.54, 20.50, 18.21, 16.90, 15.51, 15.30, 14.06, 13.99, 11.03, 10.89; ESI-MS *m/z* 786.4 [M + H]^+^.

##### N-(5-((5-bromo-1,2,3,4-tetrahydroacridin-9-yl)amino)pentyl)-olean-12-en- 28-amide E4

Brown solid (0.31 g, 0.39 mmol), yield 38.73%. ^1^H NMR (600 MHz, Chloroform-*d*) δ 9.94 (s, 1H, olean amide), 8.37 (d, *J* = 8.7 Hz, 1H, H6-Tacrine), 8.07 (d, *J* = 7.6 Hz, 1H, H8-Tacrine), 7.59 (dd, *J* = 5.8, 3.3 Hz, 1H, -NH-), 7.50 (t, *J* = 8.1 Hz, 1H, H7-Tacrine), 5.46 (d, *J* = 3.6 Hz, 1H, CH = C), 4.01 (d, *J* = 6.4 Hz, 2H), 3.42 (dd, *J* = 13.5, 6.7 Hz, 1H), 3.26 (dd, *J* = 11.5, 4.4 Hz, 1H), 3.14 (d, *J* = 5.1 Hz, 2H), 3.09 (dt, *J* = 13.2, 6.4 Hz, 1H), 2.75 (d, *J* = 6.4 Hz, 2H), 2.65 − 2.58 (m, 1H), 2.01 (t, *J* = 3.3 Hz, 4H), 1.98 − 1.93 (m, 3H), 1.83 − 1.77 (m, 2H), 1.76 − 1.70 (m, 2H), 1.68 (t, *J* = 6.9 Hz, 2H), 1.63 − 1.59 (m, 4H), 1.42 − 1.34 (m, 10H), 1.31 (s, 6H, 2CH_3_), 1.21 (s, 3H), 1.12 − 1.05 (m, 2H), 1.04 (s, 3H, CH_3_), 0.95 (d, *J* = 2.1 Hz, 6H, 2CH_3_), 0.93 (s, 3H), 0.82 (s, 3H, CH_3_), 0.80 (s, 3H, CH_3_); ^13^C NMR (151 MHz, Chloroform-*d*) δ 177.60, 166.75, 164.94, 156.30, 149.45, 143.48(C5-Tacrine), 135.56, 134.20, 133.20(C6-Tacrine), 131.40, 129.87, 128.88, 128.85, 128.45, 127.76, 124.82(C7-Tacrine), 124.55, 121.94(C8-Tacrine), 115.64, 111.93, 111.72, 77.88, 76.21, 76.00, 75.79, 67.12, 66.73, 54.38, 54.04, 47.95, 46.49, 45.66, 45.21, 42.41, 40.95, 38.31, 37.96, 37.86, 37.71, 37.69, 37.39, 35.91, 33.13, 31.97, 31.69, 31.35, 30.89, 29.67, 29.53, 29.32, 29.27, 28.66, 28.65, 28.62, 28.58, 28.33, 28.29, 28.06, 28.01, 27.95, 27.89, 27.06, 26.25, 26.18, 26.11, 24.73, 22.94, 22.93, 22.70, 22.67, 22.57, 22.44, 21.95, 21.93, 21.66, 20.50, 19.49, 17.52, 17.24, 16.09, 15.95, 14.55, 14.30, 13.09, 13.02, 11.57, 10.06, 9.93; ESI-MS *m/z* 800.4 [M + H]^+^.

##### N-(6-((5-bromo-1,2,3,4-tetrahydroacridin-9-yl)amino)hexyl)-olean-12-en-28- amide E5

Brown solid (0.27 g, 0.33 mmol), yield 33.15%. ^1^H NMR (600 MHz, Chloroform-*d*) δ 10.70 (s, 1H, olean amide), 8.29 (d, *J* = 8.7 Hz, 1H, H6-Tacrine), 7.76 − 7.69 (m, 1H, H8-Tacrine), 7.48 (ddd, *J* = 8.4, 6.9, 1.2 Hz, 1H, H7-Tacrine), 6.76 (t, *J* = 6.2 Hz, 1H, -NH-), 5.42 (d, *J* = 3.6 Hz, 1H, CH = C), 4.17 − 4.06 (m, 2H), 3.71 (tdd, *J* = 13.3, 7.6, 3.5 Hz, 10H), 3.19 (dd, *J* = 7.5, 4.2 Hz, 10H), 3.01 (t, *J* = 6.1 Hz, 2H), 2.73 − 2.67 (m, 2H), 2.63 (dd, *J* = 13.5, 4.4 Hz, 1H), 1.96 − 1.81 (m, 10H), 1.13 (s, 3H, CH_3_), 0.95 (s, 3H, CH_3_), 0.89 (d, *J* = 2.4 Hz, 6H, 2CH_3_), 0.75 (d, *J* = 20.1 Hz, 6H, 2CH_3_), 0.52 (s, 3H, CH_3_); ^13^C NMR (151 MHz, Chloroform-*d*) δ 182.30, 165.72, 157.19, 143.71(C5-Tacrine), 133.12(C6-Tacrine), 125.36, 123.56(C7-Tacrine), 119.50(C8-Tacrine), 115.69, 111.52, 56.01, 55.90, 55.78, 55.02, 51.89, 47.40, 46.52, 46.35, 43.82, 41.87, 41.64, 39.74, 39.28, 38.72, 38.66, 38.39, 36.88, 34.02, 32.97, 32.72, 32.24, 30.68, 29.71, 28.31, 28.03, 27.25, 27.07, 25.74, 23.53, 23.50, 23.41, 21.98, 20.78, 18.52, 18.20, 18.15, 17.03, 16.67, 16.61, 15.50, 15.18, 12.75; ESI-MS *m/z* 814.4 [M + H]^+^.

##### N-(2-((6-fluoro-1,2,3,4-tetrahydroacridin-9-yl)amino)ethyl)-olean-12-en-28- amide F1

Brown solid (0.17 g, 0.24 mmol), yield 24.34%. ^1^H NMR (600 MHz, DMSO-*d*_6_) δ 13.37 (s, 1H, olean amide), 8.54 (dd, *J* = 9.6, 5.5 Hz, 1H, H5-Tacrine), 7.90 (dd, *J* = 9.2, 6.2 Hz, 1H, H8-Tacrine), 7.79 (t, *J* = 5.8 Hz, 1H, -NH-), 7.49 (dd, *J* = 9.4, 2.7 Hz, 1H, H7-Tacrine), 5.12 (t, *J* = 3.7 Hz, 1H, CH = C), 4.28 (d, *J* = 5.1 Hz, 1H), 4.02 − 3.91 (m, 3H), 3.80 − 3.72 (m, 1H), 3.62 (pd, *J* = 6.6, 3.8 Hz, 2H), 3.57 − 3.51 (m, 1H), 3.42 (dt, *J* = 13.3, 7.0 Hz, 2H), 3.14 (qt, *J* = 7.4, 4.0 Hz, 2H), 3.07 − 3.01 (m, 1H), 2.97 − 2.92 (m, 3H), 2.66 (s, 2H), 1.84 (d, *J* = 6.8 Hz, 4H), 1.75 − 1.69 (m, 1H), 1.62 − 1.49 (m, 4H), 1.39 (td, *J* = 7.3, 3.4 Hz, 4H), 1.32 (dq, *J* = 8.6, 3.5 Hz, 3H), 1.01 (s, 3H, CH_3_), 0.87 (s, 3H, CH_3_), 0.85 (s, 3H, CH_3_), 0.83 (s, 3H, CH_3_), 0.63 (s, 3H, CH_3_), 0.57 (s, 3H, CH_3_), 0.13 (s, 3H, CH_3_); ^13^C NMR (151 MHz, DMSO-*d*_6_) δ 178.90, 164.89, 163.22, 162.77, 162.01, 160.59, 156.46, 144.17(C6-Tacrine), 130.11(C5-Tacrine), 122.01(C7-Tacrine), 111.70(C8-Tacrine), 77.18, 55.39, 47.28, 46.29, 45.74, 42.31, 41.53, 36.88, 36.25, 33.92, 33.83, 33.25, 33.12, 32.46, 31.23, 30.84, 29.56, 29.51, 28.64, 28.42, 27.35, 27.25, 25.95, 24.71, 23.88, 23.24, 22.68, 22.34, 21.83, 20.93, 18.55, 18.30, 17.20, 16.67, 16.41, 15.19, 12.96; ESI-MS *m/z* 698.5 [M + H]^+^.

##### N-(3-((6-fluoro-1,2,3,4-tetrahydroacridin-9-yl)amino)propyl)-olean-12-en- 28-amide F2

Brown solid (0.26 g, 37 mmol), yield 36.49%.^1^H NMR (600 MHz, DMSO-*d*_6_) δ 13.38 (s, 1H, olean amide), 8.49 (dd, *J* = 9.5, 5.5 Hz, 1H, H7-Tacrine), 7.52 − 7.48 (m, 1H, H5-Tacrine), 7.48 − 7.45 (m, 1H, H8-Tacrine), 7.41 (t, *J* = 5.8 Hz, 1H, -NH-), 5.11 (t, *J* = 3.7 Hz, 1H, CH = C), 4.29 (d, *J* = 5.0 Hz, 1H), 3.83 (q, *J* = 6.4 Hz, 2H), 3.19 (dt, *J* = 12.5, 6.3 Hz, 1H), 3.08 (dd, *J* = 13.2, 6.7 Hz, 1H), 2.95 (d, *J* = 4.8 Hz, 3H), 2.65 (s, 2H), 1.91 − 1.85 (m, 2H), 1.85 − 1.80 (m, 4H), 1.74 − 1.67 (m, 1H), 1.64 − 1.57 (m, 2H), 1.50 (d, *J* = 13.3 Hz, 1H), 1.40 (d, *J* = 9.8 Hz, 2H), 1.39 − 1.36 (m, 2H), 1.35 (s, 1H), 1.32 (d, *J* = 4.0 Hz, 1H), 1.30 − 1.20 (m, 4H), 1.04 (d, *J* = 3.1 Hz, 1H), 1.01 (s, 3H, CH_3_), 0.88 (s, 3H, CH_3_), 0.83 (d, *J* = 10.1 Hz, 6H, 2CH_3_), 0.77 (d, *J* = 13.6 Hz, 1H), 0.65 (d, *J* = 2.6 Hz, 6H, 2CH_3_), 0.59 (dd, *J* = 12.2, 1.8 Hz, 1H), 0.41 (s, 3H, CH_3_); ^13^C NMR (151 MHz, DMSO-*d*_6_) δ 177.17, 165.06, 164.87, 163.20, 162.77, 156.32, 144.45(C6-Tacrine), 121.75(C5-Tacrine), 114.89(C7-Tacrine), 111.94(C8-Tacrine), 77.20, 55.08, 47.37, 46.37, 46.33, 45.69, 41.61, 38.41, 36.91, 36.43, 36.25, 34.03, 33.33, 33.30, 32.69, 31.24, 30.85, 30.41, 28.67, 27.38, 27.33, 26.01, 23.95, 23.23, 22.71, 21.86, 20.83, 18.36, 17.09, 16.44, 15.33; ESI-MS *m/z* 712.5 [M + H]^+^.

##### N-(4-((6-fluoro-1,2,3,4-tetrahydroacridin-9-yl)amino)butyl)-olean-12-en-28- amide F3

Brown solid (0.22 g, 0.30 mmol), yield 30.28%.^1^H NMR (600 MHz, DMSO-*d*_6_) δ 13.34 (s, 1H, olean amide), 8.44 (d, *J* = 9.2 Hz, 1H, H5-Tacrine), 7.48 (m, 1H, H7-Tacrine), 7.23 (d, *J* = 5.8 Hz, 1H, H8-Tacrine), 5.10 (d, *J* = 3.8 Hz, 1H, CH = C), 4.30 (d, *J* = 5.0 Hz, 2H), 3.80 (s, 4H), 3.12 − 3.06 (m, 3H), 2.95 (d, *J* = 17.7 Hz, 6H), 2.72 (d, *J* = 13.6 Hz, 4H), 2.64 (s, 4H), 1.83 (d, *J* = 7.1 Hz, 5H), 1.66 (dd, *J* = 14.9, 7.2 Hz, 6H), 1.61 (d, *J* = 13.5 Hz, 3H), 1.45 (d, *J* = 4.5 Hz, 6H), 1.42 − 1.37 (m, 5H), 1.34 (d, *J* = 12.6 Hz, 4H), 1.23 (s, 6H), 1.03 (s, 3H, CH_3_), 0.98 (d, *J* = 13.7 Hz, 3H), 0.87 (s, 3H, CH_3_), 0.85 (s, 3H, CH_3_), 0.83 (s, 3H, CH_3_), 0.64 (d, *J* = 4.9 Hz, 6H, 2CH_3_), 0.60 (d, *J* = 11.8 Hz, 2H), 0.48 (s, 3H, CH_3_); ^13^C NMR (151 MHz, DMSO-*d*_6_) δ 176.72, 144.55, 121.70, 77.19, 55.10, 47.41, 46.45, 38.87, 38.80, 38.42, 36.91, 34.07, 33.37, 33.21, 32.84, 30.87, 29.49, 28.68, 27.97, 27.38, 26.88, 26.03, 23.97, 23.27, 22.71, 22.00, 18.35, 17.26, 16.47, 15.33; ESI-MS *m/z* 726.5 [M + H]^+^.

##### N-(5-((6-fluoro-1,2,3,4-tetrahydroacridin-9-yl)amino)pentyl)-olean-12-en- 28-amide F4

Brown solid (0.33 g, 0.45 mmol), yield 44.57%.^1^H NMR (600 MHz, DMSO-*d*_6_) δ 13.43 (s, 1H, olean amide), 8.47 (dd, *J* = 9.5, 5.5 Hz, 1H, H8-Tacrine), 7.54 − 7.50 (m, 1H, H7-Tacrine), 7.50 − 7.45 (m, 1H, H5-Tacrine), 7.21 (t, *J* = 5.7 Hz, 1H, -NH-), 5.15 (t, *J* = 3.8 Hz, 1H, CH = C), 4.29 (d, *J* = 5.1 Hz, 1H), 3.80 (d, *J* = 6.6 Hz, 2H), 3.04 (dt, *J* = 12.8, 6.4 Hz, 1H), 2.98 (d, *J* = 6.6 Hz, 1H), 2.96 − 2.94 (m, 2H), 2.76 (d, *J* = 4.6 Hz, 1H), 2.73 (s, 1H), 2.63 (s, 2H), 2.02 − 1.95 (m, 1H), 1.88 (dd, *J* = 13.9, 3.4 Hz, 1H), 1.83 (p, *J* = 3.2 Hz, 4H), 1.75 − 1.69 (m, 3H), 1.63 (t, *J* = 13.5 Hz, 2H), 1.56 − 1.49 (m, 2H), 1.40 (dd, *J* = 13.7, 5.4 Hz, 6H), 1.30 (dd, *J* = 9.8, 5.9 Hz, 3H), 1.24 − 1.21 (m, 3H), 1.15 − 1.11 (m, 1H), 1.05 (s, 3H, CH_3_), 1.02 − 0.99 (m, 1H), 0.87 (s, 3H, CH_3_), 0.85 (d, *J* = 1.6 Hz, 6H, 2CH_3_), 0.78 (d, *J* = 18.5 Hz, 1H), 0.69 (s, 3H, CH_3_), 0.67 (s, 1H), 0.62 (s, 3H, CH_3_), 0.57 (s, 3H, CH_3_); ^13^C NMR (151 MHz, DMSO-*d*_6_) δ 176.62, 164.74, 163.06, 162.78, 155.86, 144.60(C6-Tacrine), 130.11(C5-Tacrine), 121.73(C7-Tacrine), 114.92(C8-Tacrine), 114.76, 113.30, 111.92, 77.21, 70.26, 55.39, 55.15, 48.07, 47.46, 46.49, 45.67, 38.98, 38.79, 38.72, 38.44, 36.94, 36.25, 34.09, 33.38, 33.22, 32.86, 31.76, 31.24, 30.89, 29.85, 29.56, 29.50, 29.26, 29.17, 29.05, 28.76, 28.67, 27.38, 26.06, 24.19, 24.01, 23.31, 22.75, 22.57, 21.89, 20.85, 18.37, 17.31, 16.44, 15.39, 14.43; ESI-MS *m/z* 740.5 [M + H]^+^.

##### N-(6-((6-fluoro-1,2,3,4-tetrahydroacridin-9-yl)amino)hexyl)-olean-12-en-28- amide F5

Brown solid (0.24 g, 32 mmol), yield 31.81%.^1^H NMR (600 MHz, DMSO-*d*_6_) δ 13.38 (s, 1H, olean amide), 8.45 (dd, *J* = 9.3, 5.5 Hz, 1H, H7-Tacrine), 7.51 (t, *J* = 9.2 Hz, 1H, H8-Tacrine), 7.50 (d, *J* = 2.9 Hz, 1H, -NH-), 7.20 (t, *J* = 5.7 Hz, 1H, H5-Tacrine), 5.16 (t, *J* = 3.7 Hz, 1H, CH = C), 4.29 (d, *J* = 5.1 Hz, 1H), 3.81 (q, *J* = 6.9 Hz, 2H), 3.06 − 3.01 (m, 1H), 2.95 (d, *J* = 6.4 Hz, 3H), 2.76 (dd, *J* = 13.4, 4.6 Hz, 1H), 2.63 (s, 2H), 1.83 (p, *J* = 3.2 Hz, 4H), 1.71 (dq, *J* = 7.2, 3.6 Hz, 4H), 1.64 (t, *J* = 13.4 Hz, 1H), 1.54 (d, *J* = 12.4 Hz, 2H), 1.44 − 1.41 (m, 3H), 1.41 − 1.35 (m, 6H), 1.35 − 1.31 (m, 3H), 1.27 − 1.23 (m, 3H), 1.19 − 1.14 (m, 2H), 1.13 − 1.09 (m, 1H), 1.05 (s, 3H, CH_3_), 1.02 − 1.00 (m, 1H), 0.86 − 0.84 (m, 9H, 3CH_3_), 0.71 (s, 3H, CH_3_), 0.63 (t, *J* = 2.3 Hz, 1H), 0.60 (s, 3H, CH_3_), 0.59 (s, 3H, CH_3_); ^13^C NMR (151 MHz, DMSO-*d*_6_) δ 176.57, 164.80, 163.12, 155.97, 144.64(C6-Tacrine), 121.71(C5-Tacrine), 115.00(C7-Tacrine), 114.84(C8-Tacrine), 111.76, 77.19, 55.39, 55.15, 47.85, 47.48, 46.49, 45.67, 41.70, 40.92, 39.06, 38.77, 38.45, 36.95, 34.09, 33.40, 33.24, 32.88, 30.89, 30.20, 29.41, 28.64, 27.39, 26.61, 26.27, 26.08, 24.02, 23.34, 22.73, 21.86, 20.80, 18.37, 17.36, 16.41, 15.39; ESI-MS *m/z* 754.5 [M + H]^+^.

##### N-(2-((5-fluoro-1,2,3,4-tetrahydroacridin-9-yl)amino)ethyl)-olean-12-en-28- amide G1

Black solid (0.16 g, 0.23 mmol), yield 22.91%. ^1^H NMR (600 MHz, DMSO-*d*_6_) δ 7.99 (d, *J* = 8.6 Hz, 1H, H8-Tacrine), 7.95 (s, 1H, -NH-), 7.43 − 7.38 (m, 1H, H7-Tacrine), 7.29 (td, *J* = 8.1, 5.2 Hz, 1H, H6-Tacrine), 5.05 (t, *J* = 3.7 Hz, 1H, CH = C), 4.27 (d, *J* = 5.1 Hz, 1H), 3.68 − 3.55 (m, 2H), 3.39 (dq, *J* = 11.1, 5.4 Hz, 1H), 3.25 (dq, *J* = 12.9, 5.9 Hz, 1H), 2.97 − 2.94 (m, 1H), 2.91 (d, *J* = 6.3 Hz, 2H), 2.89 (s, 4H), 2.73 (d, *J* = 0.6 Hz, 4H), 1.88 (td, *J* = 13.7, 3.8 Hz, 1H), 1.81 (q, *J* = 7.4, 5.9 Hz, 4H), 1.70 (dt, *J* = 18.3, 4.1 Hz, 1H), 1.62 − 1.55 (m, 2H), 1.48 (d, *J* = 13.7 Hz, 1H), 1.40 (dt, *J* = 11.6, 5.8 Hz, 4H), 1.37 − 1.32 (m, 4H), 1.24 − 1.20 (m, 2H), 1.12 − 1.03 (m, 2H), 1.00 (s, 3H, CH_3_), 0.86 (s, 3H, CH_3_), 0.83 (d, *J* = 6.6 Hz, 6H, 2CH_3_), 0.79 − 0.73 (m, 1H), 0.65 (d, *J* = 8.7 Hz, 6H, 2CH_3_), 0.60 − 0.54 (m, 1H), 0.29 (s, 3H, CH_3_); ^13^C NMR (151 MHz, DMSO-*d*_6_) δ 177.84, 162.76, 144.29(C5-Tacrine), 123.00(C6-Tacrine), 121.93(C7-Tacrine), 120.07(C8-Tacrine), 77.25, 55.38, 55.14, 49.03, 47.41, 46.41, 45.67, 41.55, 40.84, 38.80, 38.71, 36.92, 36.24, 33.99, 33.29, 33.07, 32.47, 31.23, 30.83, 28.66, 27.38, 27.30, 26.00, 25.33, 23.93, 23.25, 22.88, 22.86, 22.49, 18.30, 16.81, 16.41, 15.31; ESI-MS *m/z* 698.5 [M + H]^+^.

##### N-(3-((5-fluoro-1,2,3,4-tetrahydroacridin-9-yl)amino)propyl)-olean-12-en- 28-amide G2

Black solid (0.20 g, 0.28 mmol), yield 28.07%. ^1^H NMR (600 MHz, DMSO-*d*_6_) δ 8.02 (d, *J* = 8.7 Hz, 1H, H8-Tacrine), 7.95 (s, 1H, -NH-), 7.49 (t, *J* = 9.2 Hz, 1H, H6-Tacrine), 7.37 − 7.32 (m, 1H, H7-Tacrine), 5.11 (d, *J* = 3.7 Hz, 1H, CH = C), 4.27 (d, *J* = 5.1 Hz, 1H), 3.59 − 3.50 (m, 2H), 3.17 (dd, *J* = 13.1, 6.4 Hz, 1H), 3.04 − 2.99 (m, 1H), 2.95 (q, *J* = 6.3, 4.7 Hz, 3H), 1.81 (d, *J* = 5.9 Hz, 4H), 1.72 (dq, *J* = 13.6, 6.5 Hz, 3H), 1.62 (dd, *J* = 12.4, 9.3 Hz, 2H), 1.51 (d, *J* = 13.8 Hz, 2H), 1.43 − 1.39 (m, 4H), 1.36 (dd, *J* = 7.9, 3.6 Hz, 3H), 1.28 (dd, *J* = 13.2, 4.1 Hz, 2H), 1.26 − 1.22 (m, 2H), 1.07 (d, *J* = 15.5 Hz, 2H), 1.01 (s, 3H, CH_3_), 0.91 (d, *J* = 13.2 Hz, 2H), 0.87 (s, 3H, CH_3_), 0.84 (d, *J* = 4.9 Hz, 6H, 2CH_3_), 0.79 (d, *J* = 13.5 Hz, 2H), 0.67 (d, *J* = 12.7 Hz, 6H, 2CH_3_), 0.61 − 0.57 (m, 1H), 0.43 (s, 3H, CH_3_); ^13^C NMR (151 MHz, DMSO-*d*_6_) δ 177.07, 165.06, 162.77, 144.46(C6-Tacrine), 123.59(C5-Tacrine), 121.77(C5-Tacrine), 120.21(C8-Tacrine), 77.25, 55.17, 47.43, 46.44, 46.29, 45.68, 41.63, 36.93, 36.68, 36.25, 34.07, 33.34, 33.30, 32.74, 31.23, 31.02, 30.85, 29.52, 28.68, 27.40, 27.35, 26.02, 25.36, 23.97, 23.27, 22.73, 22.69, 22.57, 22.18, 18.35, 17.10, 16.43, 15.36; ESI-MS *m/z* 712.5 [M + H]^+^.

##### N-(4-((5-fluoro-1,2,3,4-tetrahydroacridin-9-yl)amino)butyl)-olean-12-en-28- amide G3

Black solid (0.28 g, 0.39 mmol), yield 38.54%. ^1^H NMR (600 MHz, DMSO-*d*_6_) δ 13.24 (s, 1H, olean amide), 8.19 (d, *J* = 8.7 Hz, 1H, H8-Tacrine), 7.82 (dd, *J* = 10.8, 7.8 Hz, 1H, H6-Tacrine), 7.56 (td, *J* = 8.3, 5.3 Hz, 1H, H7-Tacrine), 7.24 (t, *J* = 5.7 Hz, 1H, -NH-), 5.11 (t, *J* = 3.7 Hz, 1H, CH = C), 4.31 − 4.27 (m, 1H), 3.84 (dq, *J* = 13.9, 6.9 Hz, 2H), 3.11 (dd, *J* = 13.1, 6.4 Hz, 1H), 3.02 (d, *J* = 4.3 Hz, 2H), 2.96 (q, *J* = 6.1 Hz, 2H), 2.75 − 2.70 (m, 1H), 2.67 (s, 1H), 2.02 − 1.95 (m, 1H), 1.69 (d, *J* = 5.2 Hz, 2H), 1.61 (t, *J* = 13.5 Hz, 2H), 1.52 (d, *J* = 14.0 Hz, 1H), 1.46 (q, *J* = 7.8, 7.2 Hz, 4H), 1.43 − 1.36 (m, 4H), 1.25 (S, 3H, CH_3_), 1.23 (s, 3H, CH_3_), 1.02 (s, 3H, CH_3_), 0.88 − 0.81 (m, 9H, 3CH_3_), 0.62 (d, *J* = 5.2 Hz, 5H), 0.61 − 0.56 (m, 1H), 0.46 (s, 3H, CH_3_); ^13^C NMR (151 MHz, DMSO-*d*_6_) δ 176.73, 155.80, 144.54(C6-Tacrine), 130.12(C5-Tacrine), 125.16(C7-Tacrine), 121.69, 112.61(C8-Tacrine), 77.18, 55.39, 55.08, 47.59, 47.40, 46.45, 45.67, 41.68, 39.22, 38.87, 38.78, 38.42, 36.89, 34.07, 33.37, 33.22, 32.82, 30.87, 29.56, 29.50, 29.17, 28.66, 28.05, 27.37, 26.88, 26.01, 23.97, 23.26, 22.71, 22.57, 21.84, 20.77, 18.29, 17.25, 16.45, 15.30, 14.43; ESI-MS *m/z* 726.5 [M + H]^+^.

##### N-(5-((5-fluoro-1,2,3,4-tetrahydroacridin-9-yl)amino)pentyl)-olean-12-en- 28-amide G4

Black solid (0.35 g, 0.47 mmol), yield 47.27%. ^1^H NMR (600 MHz, Chloroform-*d*) δ 8.23 (dd, *J* = 9.5, 5.3 Hz, 1H, H8-Tacrine), 7.53 (dd, *J* = 9.2, 2.6 Hz, 1H, H6-Tacrine), 7.17 (ddd, *J* = 10.0, 7.6, 2.6 Hz, 1H, H7-Tacrine), 6.12 (t, *J* = 5.9 Hz, 1H, -NH-), 5.37 (d, *J* = 3.7 Hz, 1H, CH = C), 3.82 (t, *J* = 7.1 Hz, 2H), 3.38 (dd, *J* = 13.6, 6.8 Hz, 1H), 3.21 (dd, *J* = 11.5, 4.3 Hz, 1H), 3.06 (dd, *J* = 13.0, 6.8 Hz, 1H), 3.02 (t, *J* = 6.2 Hz, 2H), 2.61 (t, *J* = 6.3 Hz, 2H), 2.53 (dd, *J* = 13.2, 4.3 Hz, 1H), 1.99 (td, *J* = 13.7, 3.7 Hz, 2H), 1.91 − 1.87 (m, 4H), 1.75 (t, *J* = 13.4 Hz, 1H), 1.69 − 1.62 (m, 2H), 1.56 (t, *J* = 6.3 Hz, 4H), 1.48 − 1.44 (m, 2H), 1.37 − 1.31 (m, 3H), 1.22 − 1.17 (m, 2H), 1.15 (s, 3H, CH_3_), 1.04 (dt, *J* = 13.7, 3.3 Hz, 1H), 0.98 (s, 3H, CH_3_), 0.89 (d, *J* = 1.9 Hz, 6H, 2CH_3_), 0.86 (s, 3H, CH_3_), 0.76 (s, 3H, CH_3_), 0.75 (s, 3H, CH_3_), 0.73 − 0.69 (m, 1H); ^13^C NMR (151 MHz, Chloroform-*d*) δ 178.96, 165.00, 163.30, 155.34, 152.82, 144.82(C6-Tacrine), 141.99, 141.90, 127.93(C5-Tacrine), 127.86, 123. 08(C7-Tacrine), 115.05, 114.89, 113.46, 112.10(C8-Tacrine), 106.24, 106.08, 55.20, 49.02, 47.63, 46.86, 46.45, 42.27, 42.19, 39.50, 39.13, 38.88, 38.57, 37.08, 34.27, 33.10, 32.88, 32.50, 32.06, 30.84, 30.30, 29.84, 29.79, 29.66, 29.53, 29.50, 29.48, 29.46, 29.38, 28.23, 27.42, 27.26, 25.88, 24.21, 23.96, 23.70, 23.63, 23.46, 22.83, 22.06, 21.06, 18.40, 17.13, 15.71, 15.45, 14.26; ESI-MS *m/z* 740.5 [M + H]^+^.

##### N-(6-((5-fluoro-1,2,3,4-tetrahydroacridin-9-yl)amino)hexyl)-olean-12-en-28- amide G5

Black solid (0.19 g, 0.25 mmol), yield 25.18%. ^1^H NMR (600 MHz, DMSO-*d*_6_) δ 8.08 (d, *J* = 8.8 Hz, 1H, H8-Tacrine), 7.60 (d, *J* = 9.6 Hz, 1H, H6-Tacrine), 7.48 − 7.41 (m, 1H, H7-Tacrine), 7.19 (t, *J* = 5.6 Hz, 1H, -NH-), 5.16 (t, *J* = 3.7 Hz, 1H, CH = C), 4.28 (d, *J* = 5.1 Hz, 1H), 3.65 (q, *J* = 7.0 Hz, 2H), 3.02 (dd, *J* = 13.6, 6.9 Hz, 1H), 2.99 − 2.96 (m, 2H), 2.95 − 2.92 (m, 1H), 2.75 (dd, *J* = 13.5, 4.6 Hz, 1H), 2.68 (d, *J* = 6.5 Hz, 2H), 1.82 (p, *J* = 4.0 Hz, 4H), 1.76 − 1.68 (m, 2H), 1.66 − 1.62 (m, 2H), 1.56 − 1.50 (m, 2H), 1.40 (q, *J* = 11.3, 8.8 Hz, 4H), 1.34 (d, *J* = 7.9 Hz, 2H), 1.29 (d, *J* = 7.6 Hz, 2H), 1.25 − 1.21 (m, 2H), 1.14 − 1.09 (m, 1H), 1.05 (s, 3H, CH_3_), 0.85 (d, *J* = 2.2 Hz, 9H, 3CH_3_), 0.71 (s, 3H, CH_3_), 0.60 (d, *J* = 11.8 Hz, 6H, 2CH_3_); ^13^C NMR (151 MHz, DMSO-*d*_6_) δ 176.54, 162.78, 144.64(C6-Tacrine), 124.21(C5-Tacrine), 121.73(C7-Tacrine), 121.21(C8-Tacrine), 77.21, 55.17, 48.05, 47.50, 46.50, 45.66, 41.69, 38.77, 38.46, 36.96, 34.10, 33.40, 33.23, 32.88, 30.90, 30.63, 29.47, 28.65, 27.39, 26.72, 26.42, 26.08, 24.96, 24.03, 23.34, 22.73, 22.30, 21.59, 18.36, 17.34, 16.40, 15.39; ESI-MS *m/z* 754.5 [M + H]^+^.

### Pharmacology

#### In vitro inhibition experiments of ChEs

*h*AChE, *h*BuChE, Acetylcholinesterase Activity Assay Kit and Butyrylcholinesterase Activity Assay Kit were purchased from Sigma Aldrich. And tacrine hydrochloride were purchased from MedChemExpress. The capacity of all the target compounds to inhibit *h*AChE and *h*BuChE activities were assessed by Ellman’s method. The concentration of compound producing 50% of enzyme activity inhibition (IC_50_) was calculated by nonlinear regression analysis of the response-concentration (log) curve, using the Graph-Pad Prism program package (Graph Pad Software; San Diego, CA). Results are expressed as the mean ± SD of at least three different experiments performed in triplicate.

##### Solutions preparation

Preparation of 50 mM Tris–HCl buffer solutions: 5 ml Tris solution (1 M, Ph 8.5, Beyotime Biotechnology) was dissolved in distilled water (95 ml) and adjusted with HCl to a pH of 8.0 ± 0.1. Buffer was freshly prepared and stored in the refrigerator. AChE solution 2.005 U/ml: the enzyme (271 U/mg, 0.037 mg) was dissolved in freshly prepared buffer pH 8.0 (5 ml). BChE solution 2.040 U/ml: the enzyme (7.54 U/mg, 1.353 mg) was dissolved in freshly prepared buffer pH 8.0 (5 ml). DTNB solution 3 mM: DTNB (23.8 mg) was dissolved in freshly prepared buffer pH 8.0 (20 ml) containing NaCl (116.8 mg) and MgCl2 (38.0 mg). ATChI solution 15 mM: ATChI (43.4 mg) was dissolved in distilled water (10 ml). BTChI solution 15 mM: BTChI (47.6 mg) was dissolved in distilled water (10 ml). All solutions were stored in Eppendorf caps in the refrigerator or freezer, if necessary. The pure compounds were initially dissolved in DMSO, Tacrine as standard was dissolved in distilled water. The final concentrations for the enzymatic assay were yielded by diluting the stock solution with bi-distilled water. No inhibition was detected by residual DMSO (<0.5%).

##### Enzyme Assay

A mixture of the DTNB solution (160 µL), enzyme solution (50 µL) and compounds solutions (10 µL, 3 different concentrations and once blank water) was prepared and incubated at 37 °C for 10 min. The substrate (30 µL) was added to start the enzymatic reaction. The absorbance data (l = 405 nm) was recorded under a controlled temperature of 30 °C for 15 min at 1 min intervals. All measurements were performed as triplicates.

#### Molecular docking studies

Molecular docking studies were performed using the Discovery Studio 2.0 software program (DS 2.0). The X-ray crystallographic structure of AChE (PDB code 2CKM) was obtained from the PDB (the Protein Data Bank). First, Water molecules were removed. Second, hydrogen atoms were added. Third, side chain amides and side chains bumps were fixed. The compounds B4 and D4 was imported to DS 2.0 and docked into the active site to investigate the binding modes.

#### Cell culture and MTT assay for cell viability

HepG2 and SH-SY5Y cells were obtained from the Cell Bank of Shanghai Institute of Biochemistry and Cell Biology, Chinese Academy of Sciences (Shanghai, China). HepG2 and SH-SY5Y cells were cultured in MEM or MEM/F12 supplemented with 10% FBS, 100 units mL^−1^ penicillin/streptomycin and maintained in a humidified atmosphere of 5% CO_2_ at 37 °C. Initially, 8000 cells per well were seeded in 96-well plates for HepG2 or SH-SY5Y cells, then treated with vehicle alone or tested compounds for 24 h. Then 10 *μ*L CCK8 purchased from CELLCOOK was added to each well and further incubated for another 3 h. The absorbance was measured using a microplate reader (450 nm).

#### Apoptosis detection

Annexin V-FITC and propidium iodide were used to evaluate apoptotic cells by flow cytometry. Cells were treated with different concentrations of tested compounds for 24 h. Then the cells were washed twice with phosphate-buffered saline (PBS) (centrifugation at 2000 rpm, 5 min). The collected cells were then resuspended in 500 *μ*L of binding buffer. After stained with 5 *μ*L AnnexinV-FITC and 5 *μ*L propidium iodide at room temperature for 15 min. Cells were then analysed by BD Accuri C6 flow cytometer with cell quest software (Becton & Dickinson Company, Franklin Lakes, NJ). Cells undergoing apoptosis are both Annexin V positive and PI negative.

#### Evaluation of ROS

The level of intracellular ROS was measured by using the ROS-sensitive dye, 2′,7′-dichloro-fluorescein diacetate (DCFH-DA), as a probe. In brief, cells were seeded in six-well plates at 2.0 × 10^5^ cells/well, treating with different tested compounds for 24 h, and then washed three times and incubated with final concentration of 10 *μ*M DCFH-DA for 30 min at 37 °C in the dark. After incubation, cells were washed three times and harvested in free-serum medium. The fluorescence of 2′, 7′-dichlorofluorescein (DCF) was detected by flow cytometry (488 nm excitation and 525 nm emission filters) using BD Accuri C6 flow cytometer (Becton & Dickinson Company, Franklin Lakes, NJ, USA). Data were processed by using cell quest software (Becton & Dickinson Company, Franklin Lakes, NJ).
